# ITGB1 Regulates Triple‐Negative Breast Cancer Development by Modulating the Tumor Microenvironment

**DOI:** 10.1002/advs.202513672

**Published:** 2026-02-03

**Authors:** Nuozi Song, Siqi Chen, Lei Wang, Jessica Dang, Xu Cao, Stephanie Singh, Lu Yang, Jinhui Wang, Steven T. Rosen, Yingyu Wang, Chun‐Wei D. Chen, Cheng Zhang, Mingye Feng

**Affiliations:** ^1^ Department of Immuno‐Oncology Beckman Research Institute City of Hope Duarte California USA; ^2^ Department of Pharmacology and Chemical Biology School of Medicine University of Pittsburgh Pittsburgh Pennsylvania USA; ^3^ Department of Systems Biology Beckman Research Institute City of Hope California USA; ^4^ Division of Epigenetic and Transcriptional Engineering Beckman Research Institute City of Hope California USA; ^5^ Integrative Genomics Core Beckman Research Institute City of Hope Duarte California USA; ^6^ City of Hope National Medical Center Duarte California USA; ^7^ Department of Hematology and Hematopoietic Cell Transplantation City of Hope Duarte California USA; ^8^ Beckman Research Institute City of Hope Duarte California USA; ^9^ Department of Biochemistry and Molecular Biology Mayo Clinic Rochester Minnesota USA

**Keywords:** CRISPR screen, ITGB1, R1 domain, TNBC, tumor‐associated macrophages

## Abstract

Tumorigenesis and metastasis are frequently attributed to the intricate interplay between cancer cells and the tumor microenvironment (TME). Comprehending the mechanisms and key regulators of cancer‐immune crosstalk in the TME is imperative for developing efficacious immunotherapy. Through a series of in vivo CRISPR screens, we identified tumor‐intrinsic ITGB1 as a critical regulator of triple‐negative breast cancer (TNBC) development and deciphered its underlying mechanisms. Tumoral ITGB1 facilitated the establishment of pro‐tumorigenic TME by orchestrating tumor‐associated myeloid populations. Suppressing ITGB1 favored the enrichment of anti‐tumorigenic myeloid cells and enhanced infiltration of CD4 and CD8 T cells, culminating in superior antitumor effects. CRISPR scanning pinpointed a previously unrecognized functional domain essential for ITGB1's pro‐tumorigenic activity. This domain is distinct from all known ligand‐binding sites in ITGB1. An antibody capable of sterically blocking this domain significantly impaired TNBC progression. These findings position tumoral ITGB1 as a promising therapeutic target for reprogramming the TME from a pro‐ to an anti‐tumorigenic state, thereby effectively inhibiting TNBC development. Our study uncovers a novel mechanism of TNBC development and provides a unique therapeutic strategy for targeting ITGB1 in TNBC treatment.

## 1 Introduction

Significant advancements have been made in the therapeutic landscape and prognostic assessment of breast cancer. Immunotherapy, both as a monotherapy and in combination with chemotherapy, has yielded substantial clinical benefits for breast cancer patients [1, 2, 3]. However, for highly aggressive and therapy‐resistant triple‐negative breast cancer (TNBC), only a small subset of patients experience durable responses.

A major barrier to effective treatment is the heterogeneous tumor microenvironment (TME), which plays a crucial role in TNBC pathophysiology [4, 5]. Within the TNBC TME, myeloid cells—particularly tumor‐associated macrophages (TAMs)—are the most prevalent immune cell population [6, 7, 8]. Tumor‐infiltrating myeloid cells have been implicated in tumorigenesis [9, 10, 11], metastasis [12, 13], and therapeutic resistance [7, 14, 15]. Furthermore, TAMs can suppress antitumor response from adaptive immune cells by downregulating T cell receptors, transducing negative signaling via inhibitory receptors, and instigating T cell apoptosis via death receptors [16]. Recent studies have increasingly recognized TAM reprogramming as a cancer immunotherapeutic strategy [17, 18, 19, 20]. By optimizing pharmaceutical formulations or repurposing existing therapeutics, TAM repolarization strategies have demonstrated promising efficacy in TNBC mouse models [21, 22, 23].

Cell surface receptors play critical roles in mediating the communication and interaction between cancer cells and immune cells to construct the immunosuppressive TME. Monocytes are recruited into tumor tissue via chemotactic pathways such as CSF1‐CSF1R and CCL2‐CCR2, subsequently differentiating into TAMs and adopting pro‐tumor phenotypes upon interaction with cancer cells [24, 25, 26, 27, 28]. Additionally, inhibitory receptors on macrophages, exemplified by PD1, SIRPα, and CD200R, engage self‐protective signals from cancer cells, such as PD‐L1, CD47, and CD200, rendering cancer cells the ability to re‐educate TAMs and evade immune surveillance [29, 30, 31]. Therefore, targeting cancer‐TAM interactions represents a promising approach of reshaping the immune composition of the TME and enriching tumoricidal TAMs to effectively hinder tumor progression and bolster immunotherapy efficacy [24, 29]. However, thus far, the signals or pathways mediating TNBC‐TAM interaction and modeling the landscape of the TME remain poorly characterized, representing a critical knowledge gap that limits the advancement of immunotherapeutic approaches for TNBC.

Leveraging a CRISPR‐based in vivo screening that was tailored to investigate TAM‐cancer interactions, we identified tumor‐intrinsic ITGB1 (β1 integrin) as a pivotal regulator of TNBC development. Integrin β1 forms heterodimers with a variety of integrin α subunits and is expressed on cancer, immune, or endothelial cells. The β1‐containing integrins on cancer cells have long been recognized and well‐studied for their roles in mediating interactions with the extracellular matrix (ECM), thereby activating intracellular signaling pathways to facilitate cell proliferation, migration, resistance to cell death, and maintenance of cancer stemness [32, 33, 34, 35, 36, 37, 38, 39, 40, 41]. On the other hand, ITGB1 expressed on immune and endothelial cells also plays a critical role in regulating cancer progression. For example, myeloid α4β1 integrin mediates the trafficking and aggregation of myeloid cells [38, 42]; α5β1 integrin on cancer‐associated fibroblasts (CAF) regulates their migration and promotes metastasis [43, 44, 45]; and several β1‐containing integrin heterodimers expressed on endothelial cells are essential for angiogenesis and tumor growth [43, 44, 45, 46].

Like other integrins, these traditional functions of β1‐containing integrins are primarily mediated through direct ligand engagement that transduces signals across the plasma membrane [47]. Recently, efforts have pivoted to study the function of integrins in tumor‐immune interaction. Fibroblast‐associated α5 integrin impeded T cell infiltration, and its blockade augmented the efficacy of PD‐L1 blockade [48]. Similarly, αvβ3‐integrin mediates cancer immune evasion by regulating PD‐L1 expression [49]. αvβ8 integrin regulates TGF‐β release, contributing to the construction of suppressive TME and evasion of host immunity [50, 51]. However, despite the progress, it remains unclear whether and how tumor‐intrinsic ITGB1 (tumoral ITGB1) contributes to TNBC progression by modulating tumor‐TME interactions. In addition, decades of substantial efforts have led to the development of multiple therapeutic strategies targeting β1‐containing integrins, ranging from blocking antibodies and fibronectin‐antagonizing recombinant proteins to peptide‐based inhibitors that are designed to disrupt integrin and ligand binding via active site occupancy or steric hindrance [32, 52, 53, 54]. However, clinical success has been limited, largely due to the modest efficacy of directly targeting integrin‐ECM ligand interactions, an insufficient understanding of the roles of tumoral integrins, the functional redundancy and sometimes opposing activities among different integrin subtypes, and the essential physiological functions integrins serve in normal tissues [41, 55, 56]. Advancing novel strategies targeting ITGB1 relies on a deeper understanding of its molecular mechanism in regulating tumorigenesis.

In this study, we deciphered the molecular basis of tumor‐intrinsic ITGB1 in fostering TNBC development. By integrating a CRISPR‐based domain scanning strategy with structure‐guided analysis, we unveiled the 3D functional domains of ITGB1 for mediating its TME‐modulating and pro‐tumoral roles. Notably, this critical functional domain is distinct from all its known ligand‐binding sites, and steric blockade of this domain mimicked ITGB1 deficiency in significantly suppressing TNBC tumor development. In sum, we have elucidated a novel molecular mechanism by which ITGB1 drives TNBC tumor development, providing insights that may inspire the development of innovative therapeutic strategies to inhibit TNBC tumor development.

## 2 Results

### 2.1 ITGB1 is Identified as a Critical Regulator of TNBC Tumor Development

**FIGURE 1:**
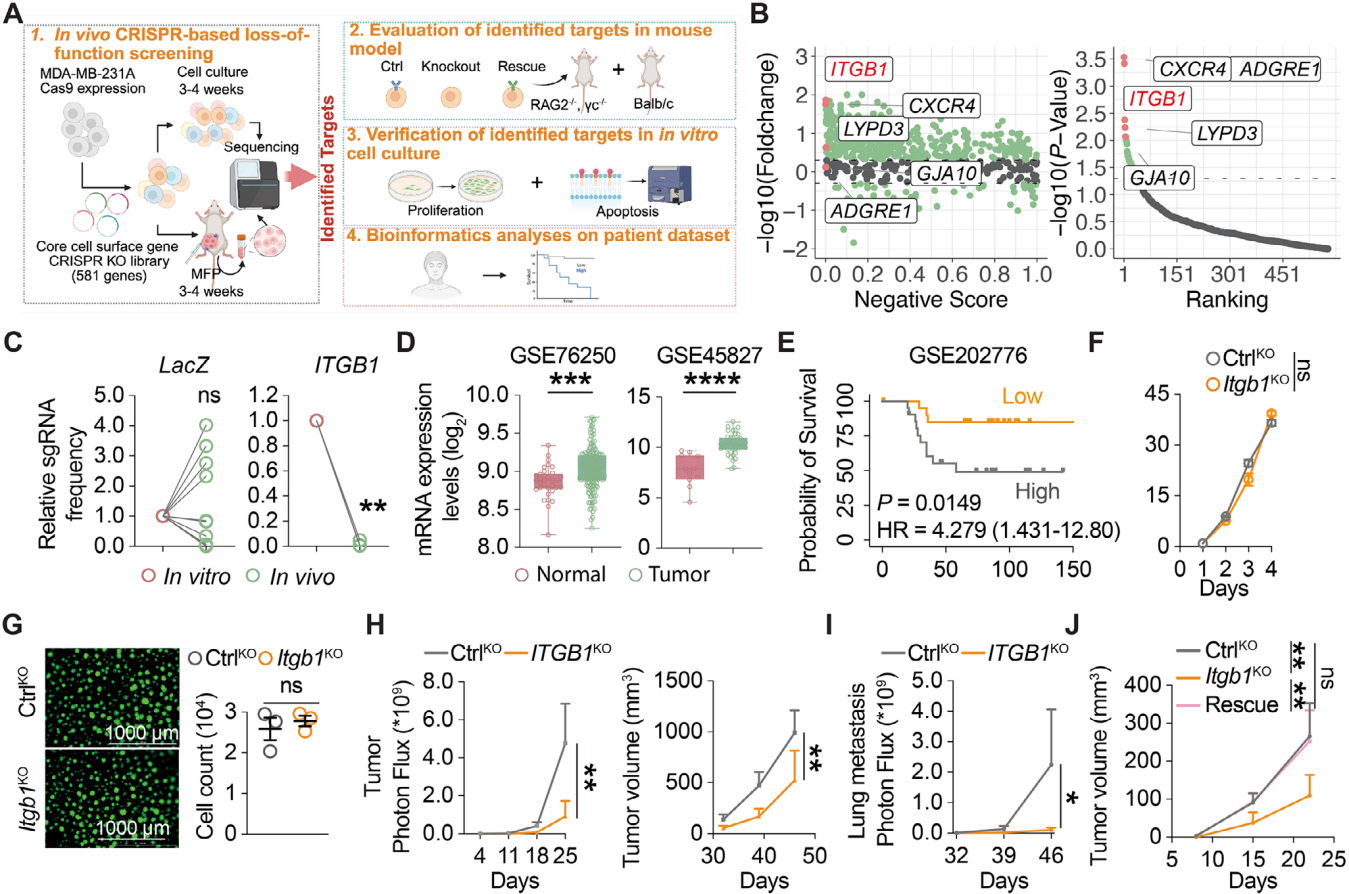
ITGB1 is identified as a critical regulator of TNBC tumor development. (A) A schematic showing the design of CRISPR screening with a sgRNA library targeting core cell surface genes and evaluation of ITGB1 function. (B) Scatter plot representing the CRISPR screen of the MDA‐MB‐231A cell, plotting ‐log10(Fold‐change) against negative score (left) or plotting ‐log10(*P*‐value) against ranking (right). The red dots represent the top 5 candidate genes with sgRNA depletion. (C) Frequencies of sgRNAs targeting *LacZ* or *ITGB1* in in vivo screened MDA‐MB‐231A cells relative to in vitro cultured cells (Ctrl). *t*‐test. (D) Expression of *ITGB1* in TNBC tissues. Unpaired *t*‐test. (E) Kaplan–Meier curves of overall survival (OS) of TNBC patients from GSE202776 by ITGB1 expression (top 20% vs. bottom 20%). (F) The proliferation of Ctrl‐KO and *Itgb1*‐KO 4T1 cells. *n* = 3; two‐way RM ANOVA. (G) Spheroid of 4T1‐GFP cells at day 7 post‐embedding in a 3D Matrigel. Representative photomicrographs and quantification of cell count. *n* = 3; unpaired *t*‐test. (H) Tumor growth in MDA‐MB‐231A‐engrafted Rag2^−/−^, γc^−/−^ mice, monitored by bioluminescence (left) imaging and tumor volume measurement (right). *n* = 6 (Ctrl‐KO) or 5 (*ITGB1*‐KO) mice; two‐way RM ANOVA. (I) Lung metastasis in MDA‐MB‐231A‐engrafted mice, monitored by bioluminescence imaging. *n* = 6 (Ctrl‐KO) or 5 (*ITGB1*‐KO) mice; two‐way RM ANOVA. (J) Growth of tumors developed by Ctrl‐KO, *Itgb1*‐KO, *ITGB1*‐Rescue 4T1 cells‐engrafted Rag2^−/−^, γc^−/−^ mice. *n* = 6 mice per group; two‐way RM ANOVA with multiple comparison test. ns, no significance, ^*^ *P* < 0.05, ^**^ *P* < 0.01, ^***^ *P* < 0.001, ^****^ *P* < 0.0001. Data are represented as mean ± SD.

We hypothesized that there are unidentified communication channels within the TME where tumor cells receive favorable signals to support tumor development under an in vivo context. For this endeavor, identification and evaluation of cell surface signals that are critical for regulating TNBC development by interacting with TME were conducted by multiple layers of experiments: 1) In vivo CRISPR‐based loss‐of‐function screening tailored for understanding TNBC‐TAM interaction. For this purpose, we utilized the Rag2^−/−^, γc^−/−^ mouse model, which lacks T, B, and NK cells but maintains functional myeloid cells [30] and allows xenografting of human TNBC cells, for the initial in vivo screening. 2) Verification of identified signals in 2D and 3D cell culture to exclude genes that are directly involved in cell proliferation and survival capacity. 3) Evaluation of identified signals in human TNBC mouse models and syngeneic immunocompetent mouse models. 4) Bioinformatics analyses on patient datasets for the correlation of identified signals with prognosis. We customized an sgRNA library targeting core cell surface genes [57], with the objective of identifying cell surface proteins facilitating TNBC tumor development in a TME‐dependent manner. The sgRNA library included 5 sgRNAs for each of the 581 genes we identified through analyzing public data deposits (a mass spectrometric cell surface protein atlas [58], a membrane protein database [59], the Human Protein Atlas (https://www.proteinatlas.org), and breast cancer cell line gene expression analyses (Table S1). The library was lentivirally delivered to Cas9‐expressing MDA‐MB‐231A single clones, a more aggressive cell line derived from human MDA‐MB‐231 TNBC cells by repetitive in vivo inoculation (Figure S1A–E). Library‐transduced tumor cells were cultured in vitro for 3 weeks to allow depletion of sgRNAs targeting genes intrinsically toxic to cell proliferation and/or survival. Tumor cells carrying the sgRNA library were orthotopically inoculated into the mammary fat pads of female Rag2^−/−^ γc^−/−^ mice, and grew in vivo for 3–4 weeks to allow sufficient screening, while in vitro cultured cells maintaining comparable library fold coverage during the same period were used as references. We reasoned that, during the selection, the frequencies of sgRNAs targeting control genes irrelevant to immune evasion should remain unchanged, while sgRNAs targeting critical signals regulating tumor development will be depleted (Figure 1A). In the screen, *ITGB1* and *CXCR4* were ranked as top candidates by the MAGeCK algorithm [60] for which sgRNA constructs were most significantly depleted in MDA‐MB‐231A cells collected from the tumors relative to the cells cultured in vitro (Figure 1B,C).

We decided to focus on *ITGB1*, which demonstrated both significant (*p* value) and the strongest (fold change) depletion in sgRNA frequencies compared to other top candidates. ITGB1 is highly expressed on TNBC cells [61]. Additionally, stimulation of CXCR4 on tumor cells has been shown to regulate the expression of β1 integrin [62], while β1 integrin ligation reciprocally regulates the expression of CXCR4 [63]. Bioinformatics analyses revealed that *ITGB1* is upregulated in TNBC tumor tissues (Figure 1D), and its higher expression is associated with poorer clinical outcome (Figure 1E). Consistently, analyses of TCGA and CPTAC datasets showed that *ITGB1* expression correlates positively with M2‐like TAM markers (CD163 and MRC1) in BRCA (Figure S1F–H). Taken together, these findings reinforce ITGB1 as a potential driver of tumor progression.

To validate the screen results, we then generated Ctrl‐KO and *ITGB1*‐KO human (MDA‐MB‐231A) and murine (4T1) TNBC cells using CRISPR gene editing (Figure S2A,B). In 2D cell culture, *ITGB1* knockout (*ITGB1*‐KO) in MDA‐MB‐231A and 4T1 cells did not alter cell proliferation or induce apoptosis (Figure 1F; Figure S2C–E). Similarly, a Matrigel‐supported 3D tumor spheroid assay revealed no discernible proliferative differences between Ctrl and *ITGB1*‐KO cells (Figure 1G; Figure S2F), in line with a previous report [64]. These findings suggested a minimum impact of ITGB1 expression or ECM‐ITGB1 interaction on intrinsic proliferation or apoptosis programs of aggressive TNBC cells. In contrast, *ITGB1*‐KO rendered orthotopic inoculated MDA‐MB‐231A cells significantly less tumorigenic (Figure 1H; Figure S2G) and metastatic (Figure 1I; Figure S2H). Consistently, in a syngeneic tumor model, *Itgb1*‐KO markedly impaired the tumor development of orthotopically inoculated murine 4T1 TNBC cells, which could be rescued by the restoration of exogenous human ITGB1 expression (Figure 1J; Figure S2I).

Integrins function in the cells as heterodimers, with 18 α and 8 β subunits forming 24 known heterodimeric integrins in humans [36]. ITGB1 pairs with multiple α subunits [39, 40], and gene expression profiling identified ITGA2, ITGA3, ITGA5, and ITGA6 as the predominant α subunits in TNBC (MDA‐MB‐231A and 4T1) tumor cells (Figure S3A). CRISPR‐mediated knockout of *ITGA2*, *ITGA5*, or *ITGA6* individually reduced tumor growth, albeit none of the individual knockouts achieved the same degree of tumor suppression as *ITGB1* knockout (Figure S3B–E). Further, dual knockout of *Itga2*, *Itga5*, or *Itga6* alongside *Itgb1* did not enhance tumor growth inhibition beyond that observed with *Itgb1* single knockout (Figure S3F). Collectively, these findings indicated that the inhibition of TNBC development by ITGB1 deficiency is solely dependent on the absence of ITGB1 and does not rely on a specific αβ1 heterodimer.

In sum, we have identified ITGB1 as a crucial facilitator for TNBC development. This pro‐tumor role of ITGB1 is independent of the intrinsic tumor cell proliferation or ECM interaction. Instead, it requires TME‐dependent stimuli.

### 2.2 ITGB1 Modulates Tumor‐Infiltrating Myeloid Cells

**FIGURE 2:**
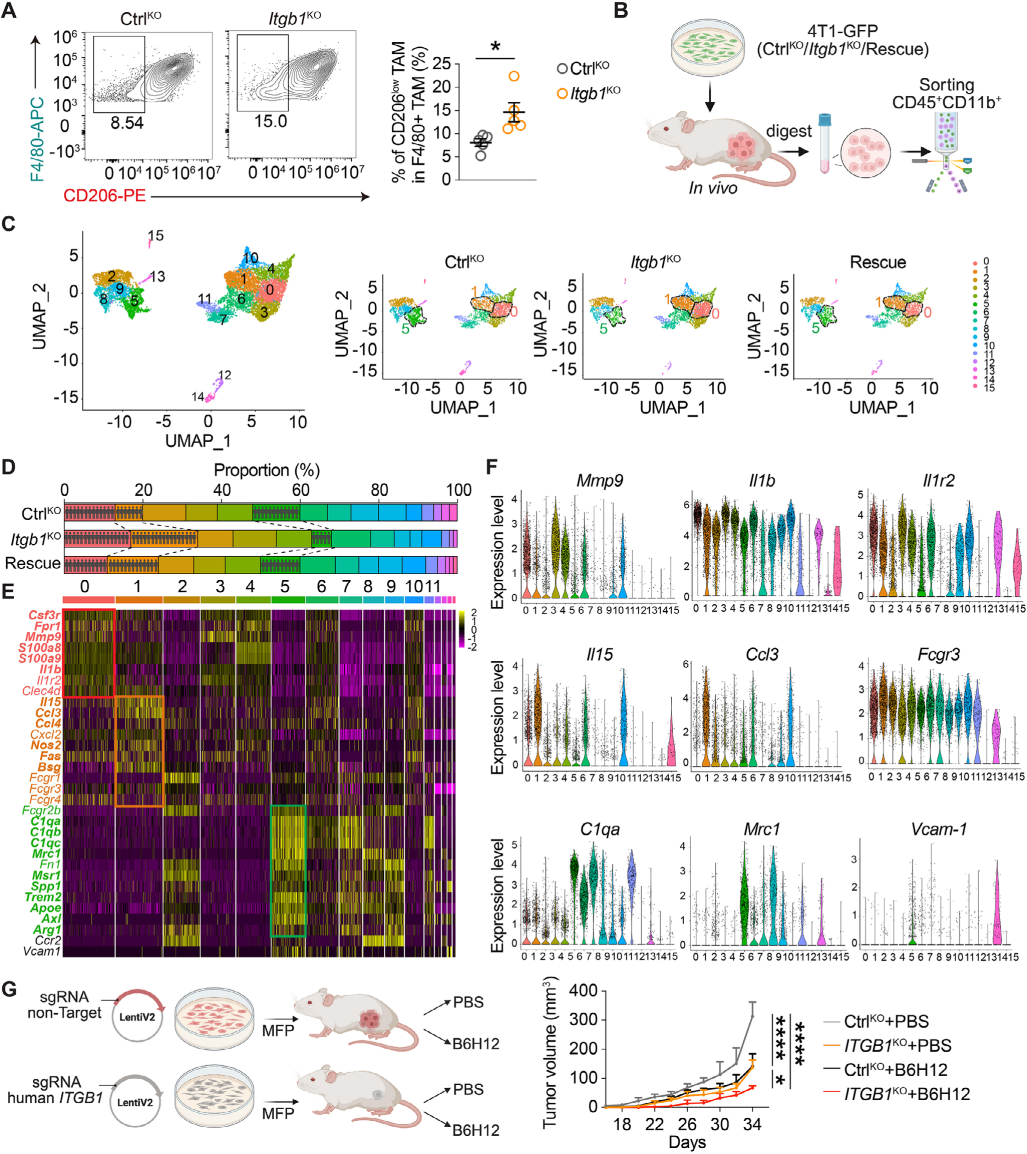
ITGB1 modulates tumor‐infiltrating myeloid cells. (A) Representative FACS plots of TAMs and quantification of CD206‐low F4/80^+^ TAMs among total TAMs (CD45^+^CD11b^+^Ly6c^−^Gr‐1^low^F4/80^+^) from Ctrl‐KO or *Itgb1*‐KO 4T1 tumor tissue. *n* = 5 mice; unpaired *t*‐test. (B) A schematic showing Single‐Cell RNA sequencing. (C) UMAP plot from scRNA‐seq data showing 16 cell clusters of the live CD45^+^CD11b^+^ compartment sorted from tumors developed by Ctrl‐KO, *Itgb1*‐KO, or *ITGB1*‐Rescue 4T1 cells‐engrafted Rag2^−/−^, γc^−/−^ mice. UMAP visualization of all identified cell clusters. Colors represent cell types (right panel). (D) Percentages of clusters in each group in panel (C). (E) Heatmap of representative genes specifically expressed in clusters 0 (*Mmp9, Il1b, Il1r2*), 1 (*Il15, Ccl3, Fcgr3*), or 5 (*C1qa, Mrc1, Vcam‐1*) from scRNAseq clustered in panel (C). (F) Expression of representative genes from scRNAseq clustered in panel (C). (G) A schematic (left) and tumor volume measurement (right) in Ctrl‐KO or *ITGB1*‐KO MDA‐MB‐231A cells engrafted mice treated with PBS or B6H12. *n* = 5 mice; two‐way RM ANOVA with multiple comparison test. ns, no significance, ^*^ *P* < 0.05, ^****^ *P* < 0.0001. Data are represented as mean ± SD.

TAMs, often the most abundant compartment in the TME, are known to be highly plastic in response to external stimuli [65, 66]. TAMs are often repolarized by tumor cells toward M2‐like pro‐tumorigenic phenotypes, thereby reciprocally promoting tumor progression [67]. To investigate how ITGB1 modulates TAM phenotype and impacts TNBC progression, we analyzed TAM composition in ITGB1‐deficient tumors. Flow cytometry analysis revealed a less immunosuppressive TME in *Itgb1*‐KO 4T1 tumors, characterized by a significant increase in anti‐tumoral CD206‐low (less immune‐inhibitory) F4/80^+^ TAMs (Figure 2A), while the proportions of the tumor‐infiltrating Ly‐6C^+^ monocytes and Gr‐1^+^ neutrophils remained comparable (Figure S4A). Similar immune‐modulatory effects were also observed in tumors derived from *ITGB1*‐KO MDA‐MB‐231A cells (Figure S4B,C).

To further dissect ITGB1's role in TAM modulation, we performed single‐cell RNA sequencing on tumor‐infiltrated myeloid cells from Ctrl‐KO, *Itgb1*‐KO, or *ITGB1*‐rescue tumors (Figure 2B). A total of 16 distinctive clusters were identified, with *Itgb1* knockout significantly expanding Clusters 0 and 1 while reducing Clusters 5, 8, and 9. Reintroducing ITGB1 reversed this phenotype, confirming a direct involvement of tumoral ITGB1 in modulating TAMs (Figure 2C–E). In line with our flow cytometry analysis (Figure 2A), *Itgb1* deficiency diminished tumor‐infiltrating monocyte/macrophage clusters 5, 8, and 9, exemplified by the expression of *Ccr2*. Clusters 5, 8, and 9 also expressed in the abundance of canonical inhibitory monocyte/macrophage genes such as *Mrc1* (encoding CD206)*, Msr1, Spp1, Trem2, Apoe, Axl*, and *Arg1* (encoding Arginase) (Figure 2E,F). Additionally, Cluster 5 expressed complement C1q‐associated genes *C1qa, C1qb*, and *C1qc*. Residing on the opposite side of the UMAP‐1 axis are the upregulated Clusters 0 and 1, which likely originated from tissue‐resident myeloid progenitors as they lack the expression of *Ccr2* (Figure 2D,F). The inflammatory phagocyte Cluster 1 was highlighted with the expression of *Il5, Ccl3, Ccl4, Nos2, Fas*, and *Bsg* (encoding CD147) (Figure 2E,F), while the inflammatory neutrophil Cluster 0 was signified by genes such as *Csf3r, Fpr1, Mmp9, S100a8/9*, and *Il1b* (Figure 2E,F).

These data indicated that ITGB1 deficiency remodeled TAMs toward less immunosuppressive phenotypes. Based on this, we hypothesized that ITGB1 knockout could enhance immunotherapy efficacy due to its TME‐modulating effects. To further interrogate its therapeutic potential, we evaluated the impact of ITGB1 deficiency on CD47‐targeted therapy, which promotes TAM‐mediated cancer cell elimination by blocking the phagocytosis immune checkpoint on tumor cells, CD47. ITGB1 knockout synergized with CD47 blockade to inhibit TNBC tumor progression (Figure 2G), suggesting that targeting ITGB1 to remodel TAMs, in combination with immune checkpoint blockade, could potentiate anti‐tumor immune responses.

Collectively, our findings establish ITGB1 as a key regulator of pro‐tumoral TAM modulation, which is pivotal for TNBC tumor development.

### 2.3 CRISPR Scanning Identifies a Key Region in ITGB1 that Mediates TAM Modulation and TNBC Tumor Development

**FIGURE 3:**
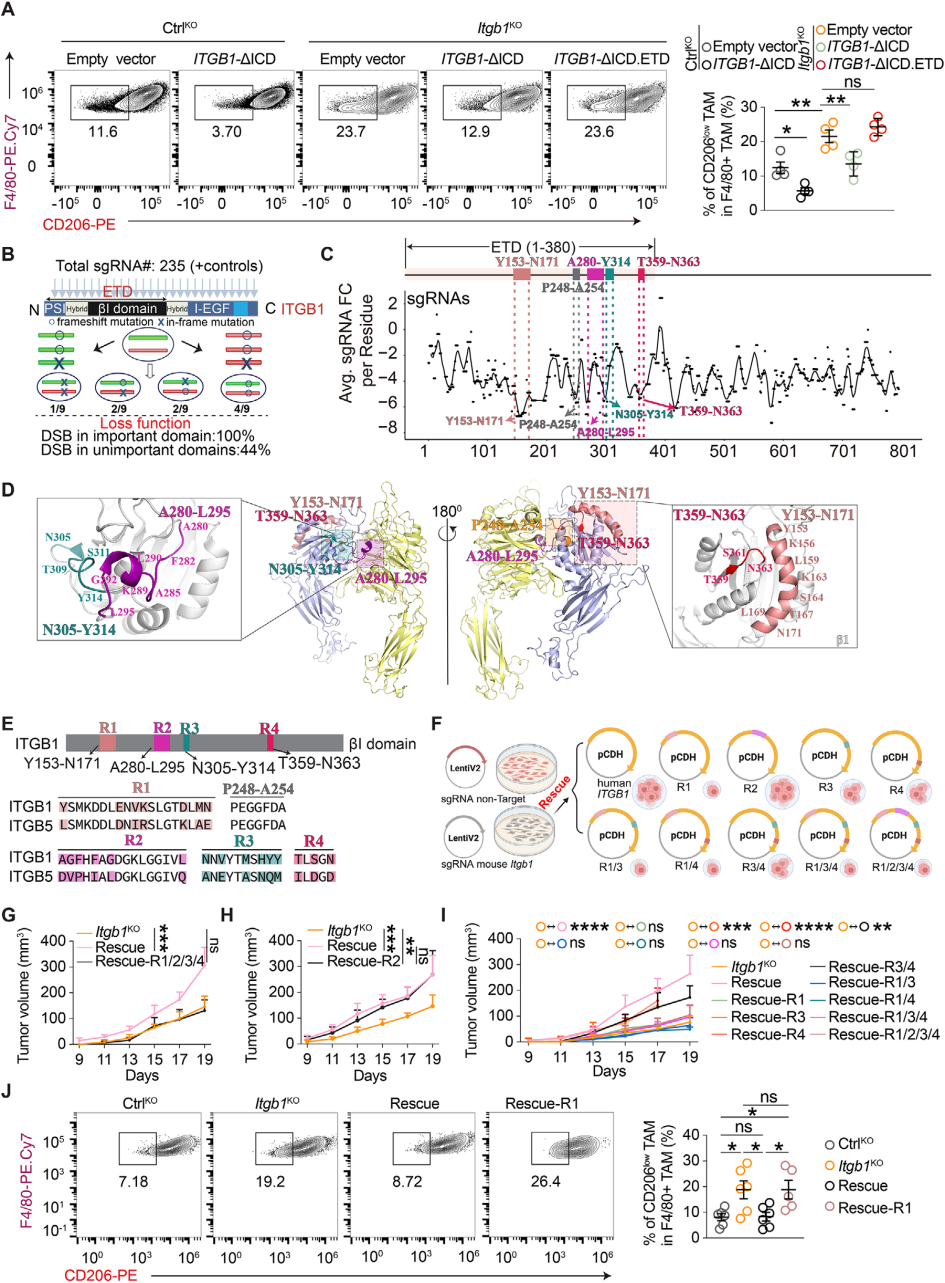
CRISPR scanning identifies a key region in ITGB1 that mediates TAM modulation and TNBC tumor development. (A) Representative FACS plots of TAMs and quantification of CD206‐low F4/80^+^ TAMs among total TAMs (CD45^+^CD11b^+^Ly6c^−^Gr‐1^low^F4/80^+^) from 4T1 tumor tissue. *n* = 4 mice; two‐way ANOVA with multiple comparison test. (B) A schematic showing the rationale and design of CRISPR scanning with a tiling sgRNA library. DNA double‐strand break (DSB) repair after Cas9 cleavage results in 1/3 of in‐frame mutations and 2/3 of frameshift mutations. In total, 4/9 (44%) of the cells receiving sgRNAs do not possess functional alleles. The chance for sgRNAs to display effects in the screens can be as low as 44% when targeting unimportant domains but up to 100% when targeting critical functional domains. (C) In vivo CRISPR screen to identify ITGB1 functional domains. Individual sgRNAs are shown by black dots and are mapped to amino acid locations. Depletion of sgRNAs was calculated based on the abundance of in vivo tumor cells and in vitro cultured control cells. The black line shows the smoothed signal levels. (D) Structure of the human α5β1 ECD (PDB ID 3VI4). α5 and β1 are colored yellow and slate, respectively. Regions Y153‐N171, P248‐A254, A280‐L295, N305‐Y314, and T359‐N363 are colored light pink, orange, deep cyan, and red, respectively. (E) Sequence alignment of ITGB1 and ITGB5 in the R1‐R4 region. (F) A schematic showing the design of region‐swapped ITGB1 mutants. (G–I) Tumor volumes measurement in 4T1‐engrafted Rag2^−/−^, γc^−/−^ mice. *n* = 6 mice (G‐H); *n* = 9 mice (I); two‐way RM ANOVA with multiple comparison test. (J) Representative FACS plots of TAMs and quantification of CD206‐low F4/80^+^ TAMs among total TAMs (CD45^+^CD11b^+^Ly6c^−^Gr‐1^low^F4/80^+^) from 4T1 tumor tissue. *n* = 6 mice; two‐way ANOVA with multiple comparison test. ns, no significance, ^*^ *P* < 0.05, ^**^ *P* < 0.01, ^***^ *P* < 0.001, ^****^ *P* < 0.0001. Data are represented as mean ± SD.

The ligand‐binding portion in the headpiece of integrin heterodimers is responsible for engaging extracellular ligands such as RGD tripeptide, laminins, or collagens [68]. Dissecting the molecular basis of ITGB1‐mediated TME modulation is critical for understanding its role in TNBC development and for developing targeted strategies to elicit antitumor TME. ITGB1 comprises major domains including an N‐terminal ectodomain (ETD, AA1–380; containing PSI, βI, and hybrid domains), a series of EGF repeats, and a C‐terminal intracellular domain (ICD). We showed that overexpression of ITGB1 or ITGB1‐ΔICD in Ctrl‐KO cells, which retain endogenous ITGB1 and intact integrin signaling, further reduced anti‐tumoral TAMs and enhanced TNBC progression (Figure 3A; Figure S5A–C), whereas ITGB1‐ΔETD (ITGB1 lacking ETD) failed to produce these effects (Figure S5D). Consistently, reintroduction of ITGB1‐ΔICD (ITGB1 lacking the ICD) in *Itgb1*‐KO cells was still sufficient to reverse the enrichment of anti‐tumoral TAM populations observed in Itgb1‐deficient tumors (Figure 3A). In contrast, further truncation of the ETD abrogated ITGB1's immunosuppressive effect (Figure 3A). Notably, overexpression of ITGB1‐ΔICD or ITGB1‐ΔETD showed no impact on cell proliferation or survival (Figure S5E,F). Consistent phenotypic and efficacious trends were also observed in MDA‐MB‐231A tumors with overexpressed full‐length ITGB1 or ITGB1‐ΔICD (Figure S5G,H). These findings indicated that TAM engagement and modulation mediated by tumoral ITGB1 depend on intercellular communication via its ETD.

Next, to further define the essential functional domains of ITGB1, we employed a CRISPR scanning strategy. Previous studies have shown that Cas9‐sgRNA‐induced in‐frame mutations occurring in critical functional domains generate more severe phenotype defects, whereas perturbations in nonfunctional domains yield milder effects [69, 70]. Thus, cell populations receiving sgRNAs targeting functional vs. nonfunctional domains of a gene perform differently in a CRISPR screen [69, 70] (Figure 3B). We designed a saturating sgRNA library targeting every protospacer adjacent motif (PAM) (NGG) site within the coding region of the *Itgb1* gene and introduced it into 4T1‐Cas9 single clones. Tumor cells carrying this tiling sgRNA library were then subjected to screening in both orthotopic xenografts and intravenous lung metastasis models.

From the CRISPR scanning, we identified five high‐ranking consensus structural regions within the ETD that potentially play important roles in TNBC development: Y153 to N171, P248‐A254, A280‐L295, N305‐Y314, T359‐N363 (Figure 3C; Figure S6A). Structurally, Y153‐N171 and T359‐N363 are located in close proximity based on the structure of human α5β1 integrin ectodomain [71], where Y153 to N171 forms a long α‐helix, and T359‐N363 forms a short loop (Figure 3D). The P248‐A254 sequence locates in the ligand‐associated metal‐binding site (LIMBS), a Ca^2^^+^ binding site that indirectly stimulates ligand binding [72, 73]. A280‐L295 is positioned in a long loop at the α5β1 interface, whereas N305‐Y314 comprises a long loop that protrudes away from α5 (Figure 3D).

Given the intricacy of structural integrity and biological function in integrins [74, 75], we employed a structural homologous region swap approach to validate the identified regions in our CRISPR scanning. The β5 integrin (ITGB5) is also highly expressed in TNBC cells (Figure S6B) and shares a high sequence and structural resemblance to β1 integrin (Figure S6C), but ITGB5 suppression or introduction of ITGB5 to ITGB1‐deficient cells showed no impact on TNBC tumor development in vivo (Figure S6D–F). Therefore, we decided to swap the identified ITGB1 regions with their structural equivalent counterparts from ITGB5 (Figure 3E), to preserve the overall structural integrity of the region‐swapped ITGB1 mutants and avoid potential irrelevant functional disruption (such as protein folding, expression, or surface exposure). The P248‐A254 segment is identical in ITGB1 and ITGB5, suggesting it is unlikely to be a key functional domain. Therefore, we focused on the four regions (R1: Y153 to N171; R2: A280‐L295; R3: N305‐Y314; R4: T359‐N363). These region‐swapped ITGB1 mutants were introduced into *Itgb1*‐KO 4T1 cells (Figure 3F) to a level comparable to endogenous ITGB1 (Figure S6G,H).

The function of the identified regions was evaluated in vivo by orthotopic inoculation of region‐swapped TNBC tumor cells to Rag2^−/−^, γc^−/−^ mice. Simultaneous swapping of all four regions completely abolished the ability of ITGB1 to rescue in vivo tumor development (Figure 3G), while the R2 region swap alone had no impact on tumor growth (Figure 3H). This prompted us to systematically evaluate region‐swapped mutants regarding R1, R3, and R4 in a combination‐exhaustion manner (Figure 3I). Notably, all mutants containing the R1 region swap abrogated the ability to rescue tumor growth, while those involving R3 and/or R4 swaps did not (Figure 3I). Phenotypically (Figure S6I), R1 region‐swap ITGB1 failed to restore the pro‐tumoral TAM population (CD206‐high), resembling *Itgb1*‐KO (Figure 3J), whereas full‐length ITGB1 rescue reinstated the pro‐tumoral phenotype of TAMs (Figure 3J), in accordance with the tumor xenograft experiments (Figure 3G–I).

Collectively, these data indicate that the Y153‐N171 (R1) region plays a critical role in modulating TAM phenotypes and mediating TNBC tumor development. Importantly, the R1 region is spatially distant from the binding interface between α and β subunits or previously identified ligand‐binding sites in β1 (e.g., RGD peptide, laminins, or collagens (collagens bind to α subunits [76]) (Figure S6J–L). This finding uncovers a previously unrecognized mechanism by which ITGB1 regulates TNBC development, independently of ECM interactions (Figure 1G; Figure S2D).

### 2.4 Steric Blocking of the R1 Region Inhibits TNBC Tumor Development In Vivo

**FIGURE 4:**
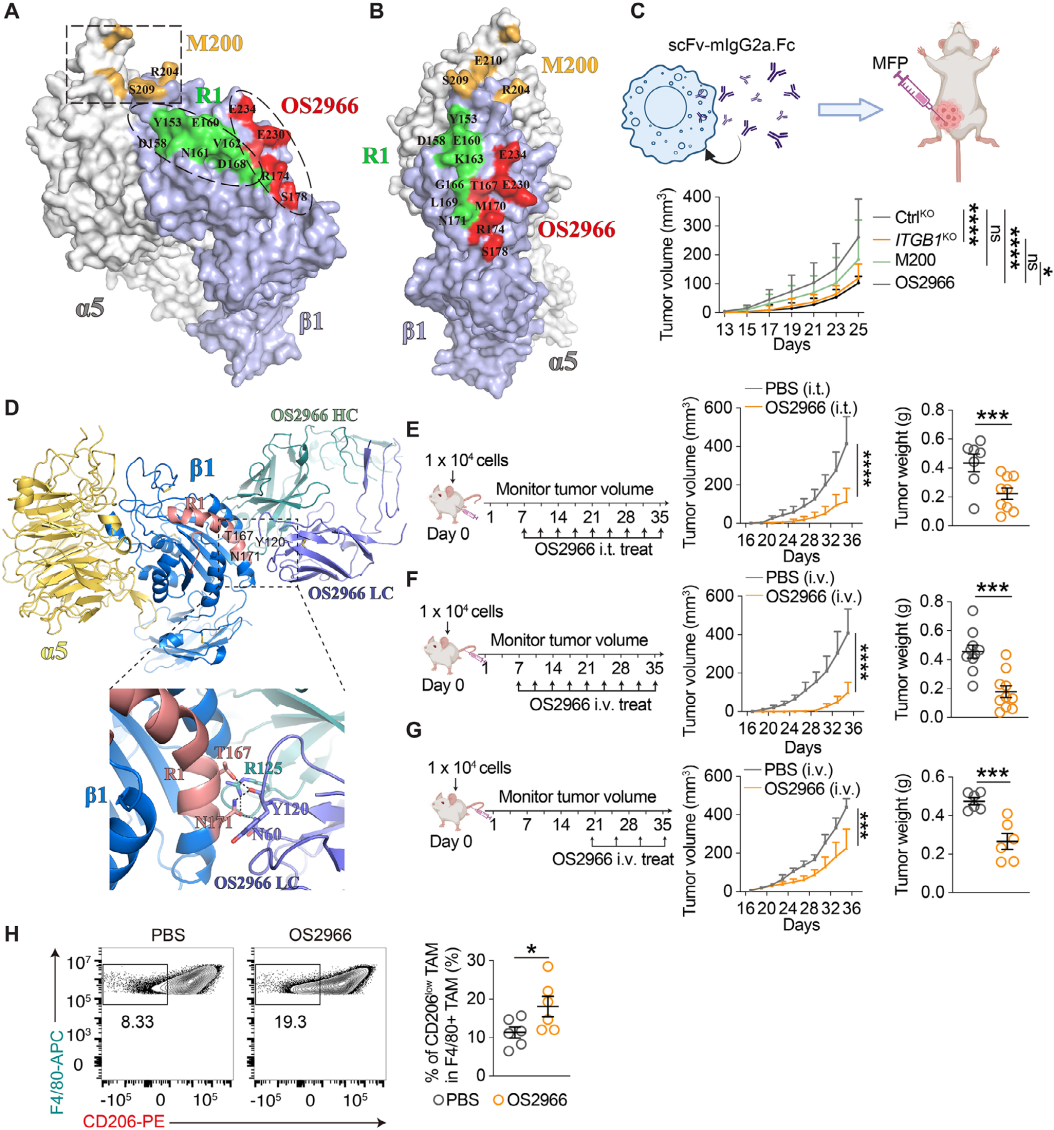
Steric blocking of the R1 region inhibits TNBC tumor development in vivo. (A,B) Structure of the human α5β1 ECD (PDB ID 3VI4). Green represents the R1 region. Yellow and red represent the areas binding with M200 or OS2966, respectively. (C) Tumor volume measurement in MDA‐MB‐231A‐engrafted mice. *n* = 6 mice; two‐way RM ANOVA with multiple comparison test (Day 25). (D) Structural model (upper) of the human α5β1 ECD in complex with OS2966 Fab. α5 and β1 are colored in yellow and blue, respectively, while the light chain (LC) and heavy chain (HC) of OS2966 Fab are colored in dark blue and light green, respectively. The interaction interface between β1 and OS2966 Fab involves a part of R1 (lower). Polar interactions are shown as dotted lines. (E–G) Tumor volume measurement in MDA‐MB‐231A‐engrafted mice treated with PBS or OS2966. *n* = 7 or 9 mice (E); *n* = 10 mice (F); *n* = 6 mice (G); two‐way RM ANOVA. (H) Representative FACS plots of TAMs and quantification of CD206‐low F4/80^+^ TAMs among total TAMs (CD45^+^CD11b^+^Ly6G^−^F4/80^+^) from 4T1 tumor tissue. *n* = 6 mice; unpaired *t*‐test. ^*^ *P* < 0.05, ^***^ *P* < 0.001, ^****^ *P* < 0.0001. Data are represented as mean ± SD.

We hypothesized that blocking the R1 region within ITGB1 would effectively disrupt ITGB1‐mediated tumor progression. Therapeutic antibodies represent a well‐established strategy for targeting cell surface receptors and modulating cell‐cell communication with delicate epitope specificity. We examined two therapeutic antibodies reported to bind to β1, volociximab (or M200) [77], and OS2966[78]. Given that the pro‐tumorigenic role of ITGB1 does not rely on a specific αβ1 heterodimer, we performed structural analysis based on α5β1, a highly expressed heterodimer in TNBC with a resolved ectodomain structure [71]. AlphaFold3 [79] was used to model the structures of M200 and OS2966 in complexes with the α5β1 ectodomain. The analysis indicated that M200 binds to α5β1, occupying the RGD ligand‐binding pocket, whereas OS2966 exclusively binds to β1, with a binding interface that substantially overlaps with R1 (Figure 4A,B; Figure S7A,B). Next, we converted M200 or OS2966 into scFv‐mIgG2a.Fc fusion proteins and evaluated their ability to inhibit TNBC progression in vivo. The Fc fragment was engineered with effector‐silencing L234A, L235A, P329G, and N297G (LALAPG.NG) mutations [80], ensuring that any observed tumor growth inhibition is attributable to direct ITGB1 blockade rather than Fc‐mediated effector function (ADCP or ADCC). MDA‐MB‐231A cells were engineered to auto‐secrete these reformatted ITGB1‐targeting antibodies, facilitating cis‐binding to tumoral ITGB1 (Figure 4C; Figure S7C,D). Importantly, OS2966 secretion resulted in a tumor inhibition effect comparable to ITGB1 knockout (Figure 4C), supporting the hypothesis that the Y153‐N171 R1 region is the key functional domain mediating TNBC tumor progression. In contrast, tumor cells secreting M200 scFv‐Fc exhibited only modest growth inhibition (Figure 4C).

To experimentally validate that OS2966 indeed binds to R1 of ITGB1 and determine its mechanism of action, we resolved a high‐resolution structure of the human α5β1 integrin headpiece in complex with the Fab region of OS2966 using cryo‐electron microscopy (cryo‐EM) (Figure S7E–G and Table S2). The structure clearly revealed that the binding interface of OS2966 directly overlaps with the R1 region but is spatially distant from the ligand‐binding site at the α5β1 interface with RGD or laminins (Figure 4D; Figure S7G). Specifically, T167 and N171 from R1 of ITGB1 form polar interactions with residues from the light chain and heavy chain of OS2966, with N171 engaging in a cation‐π interaction with Y120 of the OS2966 heavy chain (Figure 4D). These findings indicate that OS2966 can function as an ITGB1‐blocking antibody by selectively occluding the R1 region rather than interfering with classical ECM ligand interactions.

To evaluate the therapeutic potential of targeting the R1 region by steric blocking, we administered purified OS2966 antibody to TNBC tumor‐bearing mice. As expected, OS2966 showed no discernible effect on the proliferation or survival of MDA‐MB‐231A cells in vitro (Figure S7H,I), but robustly inhibited tumor growth across all experimental conditions (Figure 4E–G), irrespective of the administration route (intratumoral or intravenous) or timing (day 7 or day 21). Likewise, tumors from OS2966‐treated mice exhibited a marked enrichment of anti‐tumoral CD206‐low F4/80^+^ TAMs (Figure 4H; Figure S8A), consistent with the phenotype observed in the *ITGB1*‐KO cells (Figure S4C), indicating a shift toward a less immunosuppressive TME.

In summary, utilizing the steric hindrance, our study establishes that the ITGB1 Y153‐N171 R1 region plays a critical role in TNBC in vivo tumor development through its TME‐modulating function. Furthermore, our findings demonstrate that specific targeting of the R1 region represents a promising therapeutic strategy for TNBC intervention.

### 2.5 ITGB1 Transduces Pro‐Tumoral Signaling in a TME‐Dependent Manner

**FIGURE 5:**
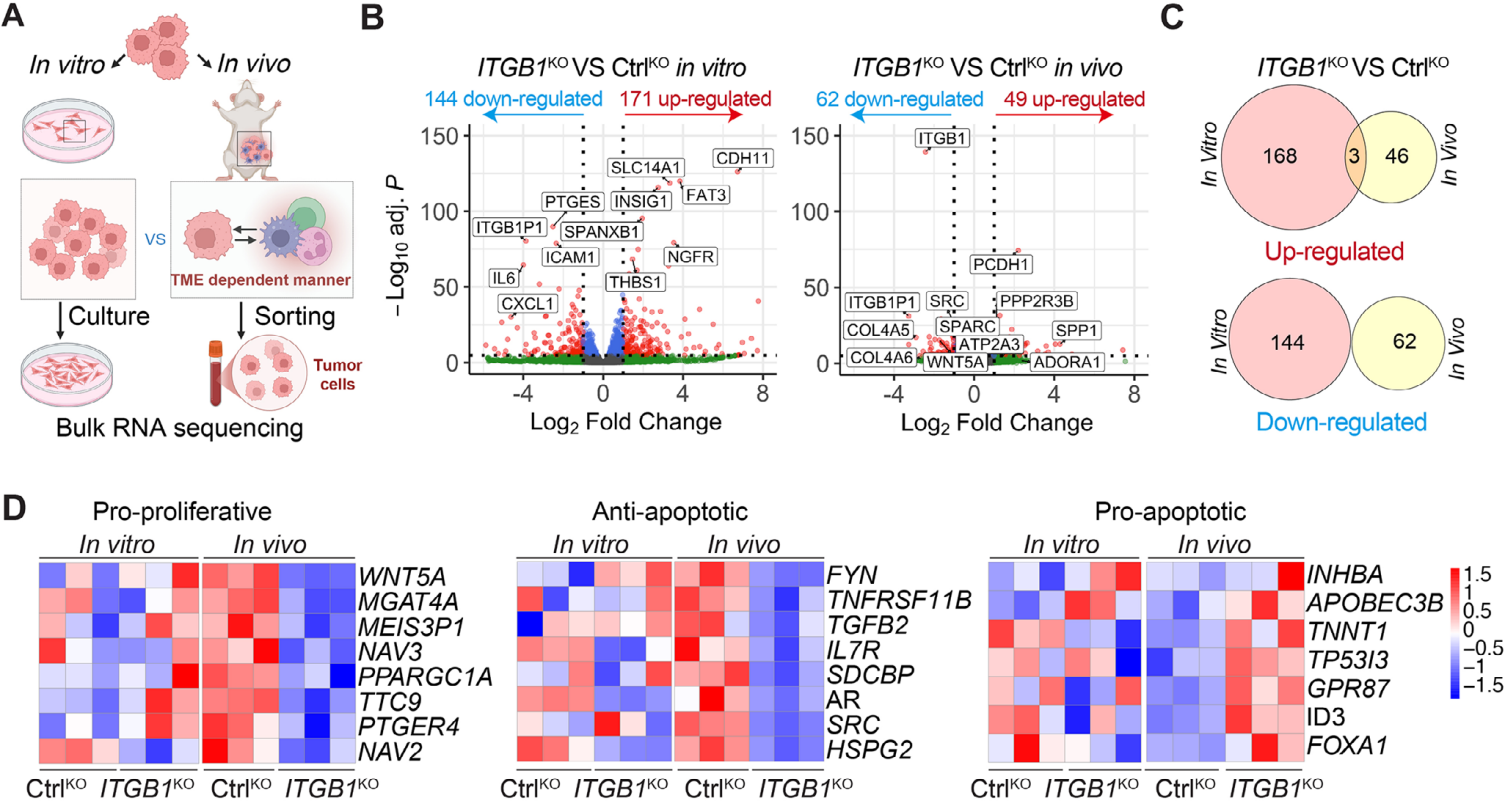
ITGB1 transduces pro‐tumoral signaling in a TME‐dependent manner. (A) A schematic showing bulk RNA‐seq of MDA‐MB‐231A cells from in vitro culture and in vivo tumor tissues. (B) Volcano plots showing differentially expressed genes between Ctrl‐KO and *ITGB1*‐KO cells in vitro (left) and in vivo (right). *P* < 10e‐5, Fold‐change > 2. (C) The Venn diagram represents the number of upregulated and downregulated genes in in vivo and in vitro conditions. (D) Heatmap visualization of pro‐proliferative, anti‐apoptotic, and pro‐apoptotic in MDA‐MB‐231A cells from in vitro culture and in vivo tumor tissues.

To dissect the signaling pathways activated by ITGB1 in a TME‐dependent context, we performed bulk RNA sequencing comparing tumor cells from in vitro cultures and in vivo xenografts (Figure 5A). We identified 315 differentially expressed genes (DEGs) in *ITGB1*‐KO cells cultured in vitro (171 upregulated, 144 downregulated) and 111 DEGs in *ITGB1*‐KO cells isolated from tumors (49 upregulated, 62 downregulated) (Figure 5B and Table S3). Interestingly, there was minimal overlap between the DEGs identified under in vitro and in vivo conditions, suggesting that ITGB1 modulated the TNBC transcriptomic landscape in response to TME‐specific cues (Figure 5C). Notably, most DEGs in in vitro cultured cells were dispersed across various unrelated pathways, whereas those from tumor xenografts converged on proliferation and apoptosis signaling, characterized by the downregulation of pro‐proliferative and anti‐apoptotic genes, alongside the upregulation of pro‐apoptotic genes (Figure 5D), suggesting that ITGB1 transduces pro‐tumorigenic signaling in a TME‐dependent manner.

### 2.6 Suppression of ITGB1 Elicits Tumor‐Specific Adaptive Immune Responses

**FIGURE 6:**
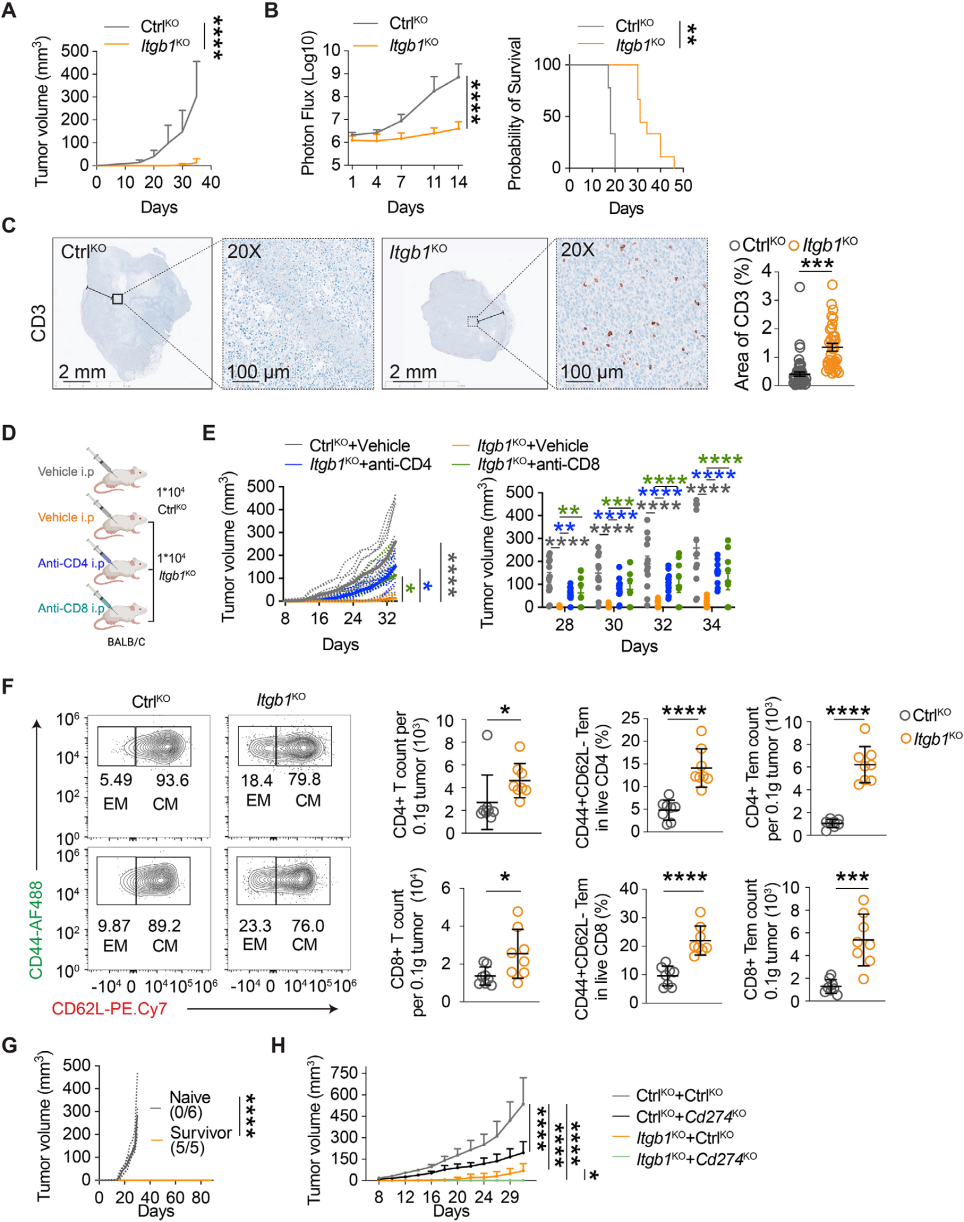
Suppression of ITGB1 elicits tumor‐specific adaptive immune responses. (A) Growth of tumors developed by Ctrl‐KO or *Itgb1*‐KO 4T1 cells in BALB/c mice. *n* = 8 (Ctrl‐KO) or 10 (*Itgb1*‐KO) mice; two‐way RM ANOVA. (B) Lung colonization (left) and survival (right) were evaluated in BALB/c mice inoculated with Ctrl‐KO or *Itgb1*‐KO 4T1 cells. *n* = 9 mice; two‐way RM ANOVA. (C) Representative photomicrographs (left) and quantification (right) of immunohistochemical staining of CD3 in 4T1 tumor tissues. *n* = 40 or 33 random fields of view from 3 mice. Unpaired *t*‐test. (D) A schematic showing the strategy of T cell depletion. (E) Growth of tumors developed by Ctrl‐KO or *Itgb1* ‐KO 4T1 cells in BALB/c mice treated with CD4‐ or CD8‐neutralizing antibodies. *n* = 12 (Ctrl‐KO + Vehicle), 12 (*Itgb1*‐KO + Vehicle), 12 (*Itgb1*‐KO + anti‐CD4), or 10 (*Itgb1*‐KO + anti‐CD8) mice; two‐way RM ANOVA with multiple comparisons. (F) Representative FACS plots and quantification of the CD4‐Tem and CD8‐Tem cells from tumor tissue. *n* = 8 mice; unpaired *t*‐test. (G) Ctrl‐KO 4T1 cells were inoculated into the contralateral sites of naïve mice or *Itgb1*‐KO 4T1 survivor mice, and tumor volume was measured. *n* = 5 or 6 mice; two‐way RM ANOVA. (H) Growth of tumors developed by Ctrl‐KO and *Cd274*‐KO 4T1‐Ctrl‐KO or *Itgb1*‐KO cells in BALB/c mice. Two‐way RM ANOVA with multiple comparisons (Day 31). *n* = 6 (Ctrl‐KO + Ctrl‐KO; *Itgb1*‐KO + Ctrl‐KO), 4 (Ctrl‐KO + *Cd274*‐KO) or 8 (*Itgb1*‐KO + *Cd274*‐KO) mice; ns, no significance, ^*^ *P* < 0.05, ^**^ *P* < 0.01, ^***^ *P* < 0.001, ^****^ *P* < 0.0001. Data are represented as mean ± SD.

Considering the pivotal role of myeloid cells in bridging innate and adaptive immunity [81, 82, 83], it is imperative to investigate whether and how the impact of tumoral ITGB1 on myeloid phenotype could lead to anti‐tumor adaptive immune responses. To this end, we examined how ITGB1 deficiency impacts TNBC tumorigenesis within an immunocompetent environment. Orthotopically inoculation of *Itgb1*‐KO 4T1 in immunocompetent BALB/c mice resulted in markedly impaired tumor development, compared to Ctrl‐KO cells (Figure 6A; Figure S9A). Of note, in contrast to tumor growth in immunodeficient mice (Figure 1H–J; Figure S3D–F), where *Itgb1*‐KO tumors displayed delayed but not abrogated progression, *Itgb1*‐KO 4T1 tumors were largely rejected in immunocompetent mice. Similarly, in a lung metastatic model, intravenously inoculated *Itgb1*‐KO 4T1 cells demonstrated significantly compromised capacity to colonize distant organs compared to Ctrl‐KO cells, which translated to substantially improved survival outcomes (Figure 6B; Figure S9B).

To determine whether ITGB1 ablation in TNBC cells sensitized the TME, we first performed immunohistochemical staining of tumor tissues, revealing significantly increased T cell infiltration in the kernel region of *Itgb1*‐KO tumor tissues (Figure 6C; Figure S9C). To further phenotypically characterize T cell subsets contributing to the tumor rejection, we employed neutralizing antibodies to deplete CD4^+^ or CD8^+^ T cells (Figure 6D; Figure S9D). Depletion of either T cell subset attenuated the *Itgb1*‐KO tumor rejection and restored tumor growth (Figure 6E; Figure S9E,F). Flow cytometry demonstrated a significant increase in tumor‐infiltrating CD4^+^ and CD8^+^ T cells, whereas Gr1^+^ neutrophils and NK1.1^+^ NK cells remained unchanged in the *Itgb1*‐KO tumor tissues (Figure 6F; Figure S10A,B). TAM profiling revealed that *Itgb1*‐KO increased the frequency of CLEC4D^+^ and FCGR4^+^ TAMs while decreasing TREM2^+^ and CD204^+^ subsets (Figure S10C), consistent with the major macrophage population shifts identified in our scRNA‐seq analysis. Moreover, within the tumor‐infiltrated CD4^+^ and CD8^+^ populations, we observed elevated proportions and absolute counts of effector memory (CD44^+^ CD62L^−^) T cells in both helper and cytotoxic subsets (Figure 6F). To assess the functionality of the enhanced effector memory phenotype in tumor‐infiltrated lymphocytes, we conducted a tumor rechallenge experiment (Figure S11A). All BALB/c mice that had remained tumor‐free following primary *Itgb1*‐KO 4T1 tumor rejection exhibited complete resistance to rechallenge with parental 4T1 cells on the contralateral side, whereas all age‐ and sex‐matched naïve controls developed tumors, indicating the establishment of durable anti‐tumor immunity by ITGB1 deficiency (Figure 6G; Figure S11B,C). Given the observed enhancement of adaptive immunity upon ITGB1 loss, we next explored whether targeting ITGB1 could cooperate with immune checkpoint blockade to further amplify anti‐tumor responses. Notably, double knockout of *Itgb1* and *Cd274* (encoding PD‐L1) in 4T1 cells resulted in markedly enhanced tumor suppression compared to single knockouts, suggesting the therapeutic potential of ITGB1 targeting in synergy with immune checkpoint inhibition (Figure 6H; Figure S11D). Lastly, we hypothesized that exogenous expression of ITGB1 ETD domain, lacking transmembrane and cytosolic regions necessary for outside‐in signaling to facilitate tumor survival, may act as a competitive antagonist to endogenous ITGB1 on TNBC by interfering with its interaction with TAMs. To test this, 4T1 cells were engineered to auto‐secret WT or R1‐mutated ITGB1 ETD and orthotopically inoculated into BALB/c mice. Indeed, secretion of WT ETD significantly inhibited tumor growth, whereas the mutation of the R1 region completely abrogated this tumor‐inhibitory effect (Figure S11E).

## 3 Discussion

**FIGURE 7:**
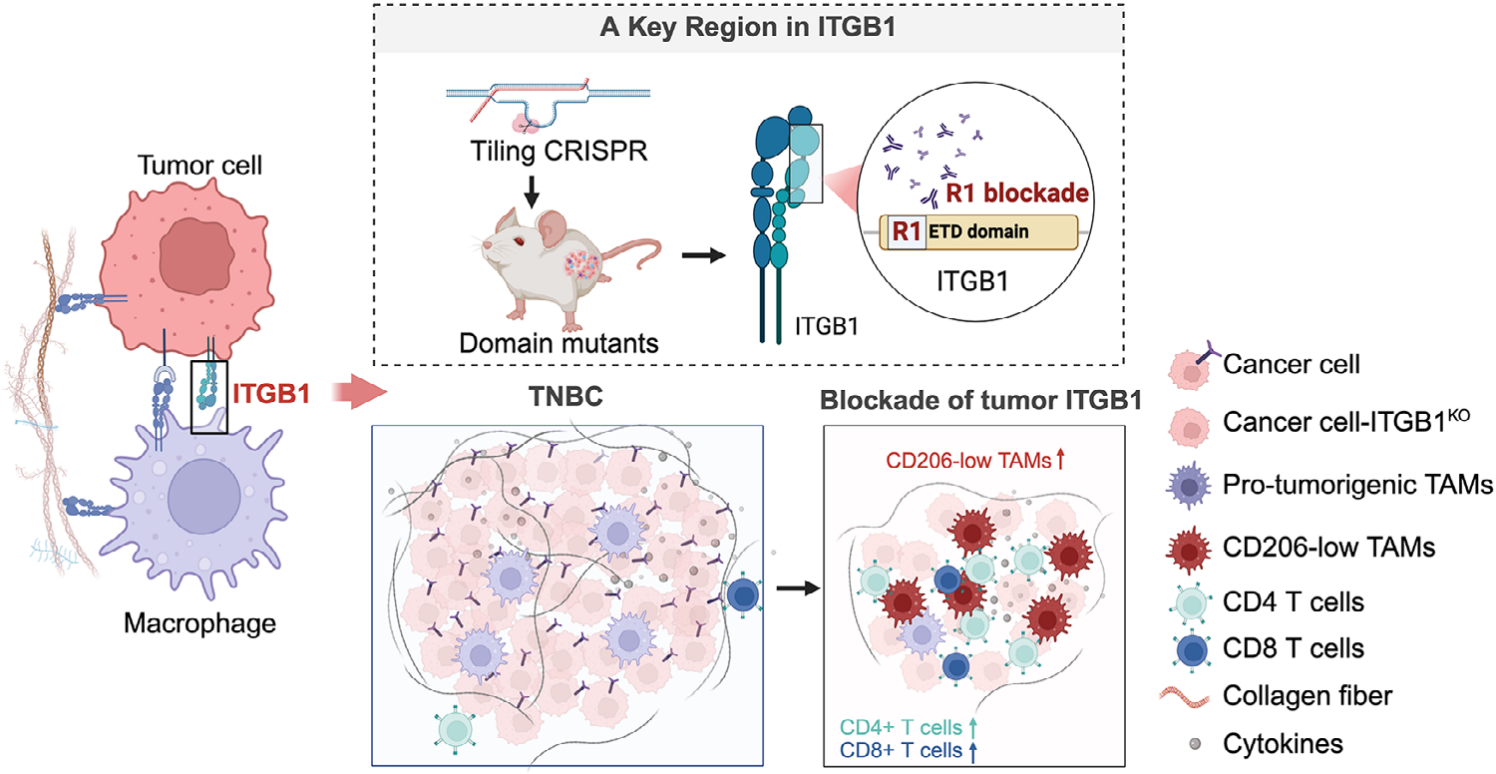
The impact of ITGB1 on tumor microenvironment and tumor development in TNBC. Tumor intrinsic ITGB1 promotes tumor progression through a previously unrecognized mechanism that is independent of its canonical ligand‐binding functions. Instead, this activity is mediated by the R1 region, which is distant from known ligand‐binding domains within ITGB1. ITGB1 orchestrates immunosuppressive remodeling of the TME by modulating the TAM phenotype, which is dependent on the R1 region. Targeted disruption of this R1 region, through genetic mutations or steric blocking by an ITGB1‐specific antibody (OS2966), significantly suppressed TNBC development.

TAMs are often the most abundant immune cell population within solid tumors, including TNBC. However, their roles and functions in tumor progression remain incompletely defined, and at times controversial, owing to the complexity and dynamic nature of TAM populations. Targeting TAM‐tumor interactions holds significant therapeutic promise, yet effective clinical translation requires a detailed mechanistic understanding of these interactions and identification of key regulators. A central theme of our research is to understand how macrophages sense, interact with, and respond to tumor cells, and how these interactions can be exploited therapeutically [84, 85, 86]. In this study, as a continuation of this broader effort, we performed an unbiased in vivo screen to identify tumor‐derived surface molecules that shape macrophage behavior during TNBC progression. We identified ITGB1 as a key driver of TNBC tumor development, promoting TAM‐mediated immune suppression and transducing pro‐tumorigenic signaling (Figure 7). Importantly, we uncovered a previously unrecognized region in ITGB1, distinct from its classical ligand‐binding domains, as a critical mediator of ITGB1's TME‐dependent pro‐tumoral function. This discovery opens new avenues for therapeutic targeting of ITGB1 in TNBC.

CRISPR‐Cas9 screening with genome‐wide or customized sgRNA libraries has identified numerous genes and signaling pathways as critical facilitators of cancer development or immunotherapy targets, including mTOR and Hippo signaling [87], MGA [88], LGALS2 [89], SNRPC [90], and CDK7 [91]. Additionally, a genome‐wide in vivo CRISPR screening identified genes that sensitize TNBC to paclitaxel treatment [92]. Our work focused on identifying TNBC tumorigenesis facilitators that specifically function in response to in vivo stimuli by screening a custom cell surface protein‐focused sgRNA library, and identified ITGB1 as a major driver for the TNBC tumor development in vivo.

Traditionally, domain truncation and amino acid scanning mutation were heavily relied on for functional domain mapping and epitope characterization [69, 93, 94]. Here, our study innovatively combined CRISPR‐scanning and cryo‐EM structural analysis, leading to the identification of a previously uncharacterized functional region in β1 integrin that governs TNBC tumorigenesis in vivo. Further functional validation was achieved by swapping this region with a structurally homologous but functionally distinct β5 integrin domain, underscoring the critical role of ITGB1 structural integrity in mechanotransduction and biochemical signaling [74, 95]. The precise identification of 3D functional domains that mediate ITGB1‐TME interactions provides a rational framework for developing therapeutic strategies targeting these critical regions for TNBC treatment.

Various β1 integrin targeting antibodies have been developed. For instance, HUTS‐4 recognizes β1 residues E371 and K417 which are exposed in its open conformation [96, 97]; TS2/16 targets the α2 helix in the βI domain, engaging residues N207, K208, and V211^99^; and SG/19 binds the interface of the I‐like and hybrid domains via residues R98, S99, T102, K105, R174, R175, S178, S419 and I420^73^. Among these, OS2966 binds exclusively to β1 integrin, whose binding interface is away from the well‐characterized heterodimerization interface or ligand‐binding sites. OS2966 uniquely binds to β1 integrin through residues T167 and N171, sterically hindering the Y153 to N171 R1 region, which we identified as essential for TNBC progression. Indeed, OS2966 more potently inhibited in vivo TNBC tumor development compared to antibodies targeting classical ECM‐binding sites, suggesting that targeting the unconventional R1 region (Y153‐N171) may offer a more effective therapeutic strategy.

Integrins transmit signals bidirectionally across the plasma membrane through coordinated inside‐out and outside‐in mechanisms [41]. Upstream activation is triggered by surface chemokine receptor‐ or GPCR‐induced RAP1‐RIAM‐talin pathways, in which talin and kindlin sequentially bind the β‐subunit to convert integrins from a bent, inactive to an extended, high‐affinity state. Negative regulators (e.g., SHARPIN, ICAP1) maintain basal inhibition, while actin tension and α‐actinin‐talin cooperation stabilize activation [98]. Activation causes conformational changes in integrins, enabling high‐affinity binding with their ligands. Downstream, ligand engagement and clustering recruit FAK, SRC, and ILK, propagating MAPK/ERK, PI3K‐AKT, JNK, and NF‐κB cascades that control adhesion turnover, cytoskeletal remodeling, and survival. Mechanical loading further activates YAP/TAZ and MRTF‐SRF transcriptional programs [99, 100].

Structurally, the extracellular domain of β integrins consists of a βI domain linked through the hybrid domain to the PSI domain, four EGF‐like repeats, and the β‐tail domain, together forming a flexible stalk. Among them, the βI domain harbors the Metal Ion‐Dependent Adhesion domains (MIDAS), which mediate direct binding to ECM ligands such as fibronectin, collagen, and laminin, and along with Adjacent to MIDAS (ADMIDAS) and Ligand‐Associated Metal‐Binding Site (LIMBS), which regulate the conformation and ligand binding affinity [72, 73, 101]. Of ITGB1, the Asp130, Ser134, Asp137, and Asp138 of the α1‐helix are important residues interacting with ECM ligands [102]. Of note, the R1 region (Y153‐N171) sits on the far side of the ECM ligand binding sites as well as the α integrin heterodimer interface. Given that the R1 region (Y153‐N171) does not participate in known integrin‐ligand interactions, it is possible that this region modulates TNBC progression via ligand‐independent mechanisms, a hypothesis warranting further investigation.

Targeting integrins and their associated signaling pathways has been proposed as a promising strategy for cancer treatment [32, 54, 103, 104]. However, despite the rationale behind these approaches, integrin‐targeting agents have yielded limited clinical benefits [55, 56]. Several factors may contribute to these challenges [55, 56, 105]. First, the existence of multiple integrin heterodimers with redundant functions likely enables compensatory signaling, reducing the efficacy of therapies targeting a single heterodimer. Second, the essential physiological functions of integrins, as exemplified by embryonic lethality in *Itgb1*‐knockout mice [106], may raise potential toxicities in normal tissue cells when integrins are targeted, thereby constraining the therapeutic window of integrin antagonists. Third, inhibitors designed to prevent ligand binding may not be capable of fully abrogating pro‐survival signaling or could potentially induce conformational changes that favor signal transduction. Selectively targeting pro‐tumor functions of β1 integrin while sparing its role in normal tissues remains a critical challenge. Our identification of a previously unrecognized domain (R1 region) within β1 integrin represents a potential breakthrough in this regard. Given that β1 integrin forms heterodimers with multiple α subunits in cancer cells, targeting the β1 integrin R1 region may circumvent the need for broad‐spectrum integrin inhibition, thereby overcoming functional redundancy. Furthermore, our findings, with OS2966 as proof of concept, demonstrate that specifically disrupting the R1 region elicits superior anticancer effects in TNBC, comparable to the genetic knockout of ITGB1. These results pave the way for the future development of more effective ITGB1‐targeting inhibitors for TNBC treatment. Notably, targeting a region distant from known ligand‐binding sites may enable modulation of the TME while preserving integrins’ intrinsic physiological functions in normal tissue cells. This approach offers a novel strategy for cancer therapy with heightened efficacy and reduced on‐target‐off‐tumor toxicity. We demonstrated that β1 integrin deficiency promoted T cell infiltration, a feature associated with improved survival and enhanced response to therapies in TNBC patients [107, 108]. Our discovery supports the notion that macrophages serve as intermediaries through which ITGB1 influences broader antitumor immunity.

Of note, although our analyses indicate that the R1 region likely adopts a conformationally stable architecture, ITGB1‐mediated tumor‐macrophage interactions may nevertheless be modulated by additional factors within the TME. Furthermore, this study has not characterized the macrophage receptor(s) that directly interact with the R1 region of β1 integrin. Elucidating additional TME‐associated modulators and defining the macrophage receptor(s) engaging the R1 region, as well as determining how these interactions are shaped by microenvironmental context, warrants future effort.

Our work uncovered a TME‐dependent regulatory mechanism of ITGB1 that converged on anti‐apoptotic signaling in TNBC cells. Further investigation is warranted to elucidate the source and mechanism of how such pro‐tumoral anti‐apoptotic signaling is mediated by ITGB1 in tumors. Previous studies investigating integrins’ role in cell proliferation, adhesion, and activation of signaling pathways such as SRC/FAK/ERK have yielded varying results across different in vitro models [41, 75]. Knockdown of ITGB1 using siRNA has been reported to reduce phosphorylation of SRC, FAK, and AKT [109, 110, 111], whereas other studies have observed enhanced EGFR signaling, increased ERK signal transduction, or unaltered FAK, ERK, and AKT activity following ITGB1 knockdown or α5β1 inhibition [112, 113]. These disparities may arise from differences in cell types, culture conditions, or gene knockdown approaches. Furthermore, short‐term gene knockdown may yield distinct effects compared to sustained long‐term suppression [112]. Importantly, in vitro observations frequently fail to recapitulate in vivo effects within the intricate TME [114, 115]. Our study demonstrated that while the aggressive TNBC cells exhibited minimal reliance on SRC/ERK/Akt signaling under in vitro conditions, ITGB1‐mediated cancer‐TME interaction elicited the activation of these pathways in vivo, playing a pivotal role in TNBC tumor development.

Our discovery has undergone rigorous validation across multiple TNBC models, including both human and mouse TNBC lines in immunodeficient mice that only possess functional myeloid cells and in immunocompetent mouse models. A highly malignant TNBC cell line derived from the widely used MDA‐MB‐231 was utilized for initial screening, followed by functional validation in syngeneic murine models. Consistent results were obtained in 4T1 mouse TNBC cells within immunocompetent mice, further underscoring the critical roles of ITGB1 in breast cancer tumorigenicity. However, our study was performed solely on mouse models generated by tumor cell lines inoculated on in‐bred mice strains. Investigations carried out on humanized mouse models incorporating patient specimens (PDX) would further address inter‐patient and intra‐tumoral heterogeneity and bridge the gap between bench and bedside. Additionally, while our work pointed a new direction in advancing ITGB1‐targeted therapeutic development, the identification of an effective therapeutic modality to precisely target the R1 region of ITGB1 in TNBC patients requires further examination, in addition to the safety and therapeutic efficacy validation, to support clinical translation.

## 4 Methods

### 4.1 Cell Lines

The MDA‐MB‐231 and 4T1 cell lines were purchased from the American Type Culture Collection (ATCC). The MDA‐MB‐231 and MDA‐MB‐231A cells were cultured in Dulbecco's Modified Eagle Medium (DMEM) (Gibco), supplemented with 10% fetal bovine serum (FBS) (Gibco) and 1% penicillin/streptomycin. 4T1 cells were maintained in RPMI 1640 (Gibco) enriched with 10% FBS (Gibco) and 1% penicillin/streptomycin. Both *ITGB1*‐KO MDA‐MB‐231 and *Itgb1*‐KO 4T1 cells, as well as hITGB1 rescue 4T1 cells, underwent lentiviral transduction and were subsequently FACS‐sorted and maintained in culture. The expression and purity of mITGB1 and hITGB1 were routinely verified. All cells were cultured at 37°C in a 5% CO_2_ atmosphere. Emphasis was placed on utilizing low‐passage cells, with large batches from below the third passage being cryopreserved. This study exclusively employed cells with passage numbers under 20. Furthermore, every two months, the cells underwent mycoplasma contamination testing as part of standard quality control.

### 4.2 Mouse

Mice were housed at the City of Hope Animal Facility under the following conditions: a 12‐h light/12‐h dark cycle, temperatures maintained between ∼18°C and 23°C, and a relative air humidity of 40%–60%. Before initiating the cancer‐related experiments, the mice exhibited good health and were assessed as BAR (bright, alert, and responsive). Their health was monitored daily. Female mice, aged between 8 and 12 weeks, were utilized for the experiments. Rag2^−/−^, γc^−/−^ mice, and BALB/c mice were bred at the Animal Resources Center of the City of Hope Comprehensive Cancer Center. The Rag2^−/−^, γc^−/−^ mouse strain was kindly provided by Dr. Irving L. Weissman from Stanford University. All animal experiments were approved by the Institutional Animal Care and Use Committee (IACUC) of City of Hope, approval numbers IACUC#17051 and #23046, and were conducted in accordance with institutional guidelines and national regulations.

### 4.3 In Vivo CRISPR Screen

For the cell surface proteome CRISPR library, 2905 sgRNA sequences targeting 581 cell surface protein genes and 68 control sgRNA sequences were designed. Guide RNA oligos were synthesized by microarray (CustomArray; for library cloning) and cloned into the ipUSEPR lentiviral sgRNA vector [116, 117, 118] using the BsmBI (NEB) restriction sites. For the CRISPR tiling library, 235 sgRNA sequences targeting the coding regions of mouse ITGB1 (Table S1) and 99 control sgRNA sequences were designed. Guide RNA oligos were synthesized by microarray (CustomArray; for library cloning) and cloned into the pLKO5.sgRNA.EFS.tRFP.Puro vector using the BsmBI (NEB) restriction sites. The pLKO5.sgRNA.EFS.tRFP.Puro is generated based on pLKO5.sgRNA.EFS.tRFP vector, a gift from B. Ebert (Addgene, plasmid #57823) [119], and the puromycin N‐acetyl‐transferase was cloned in to provide puromycin resistance.

The cloned libraries were quality‐checked via NextSeq sequencing, packed into lentivirus, and transduced into MDA‐MB‐231A or 4T1 Cas9‐expressing single‐cell clones at MOI = 0.2 to ensure single‐copy sgRNA lentiviral transduction. Transduced cells harboring the CRISPR library were cultured in vitro for 3 weeks to pre‐deplete lethal sgRNAs impacting cell survival and proliferation. These cells were then inoculated into the mammary fat pads of Rag2‐/‐, γc‐/‐ mice and retrieved 3–4 weeks after engraftment. The unique sequence of each sgRNA was amplified, and the frequencies of sgRNA constructs were analyzed using high‐throughput sequencing followed by the MAGeCK [60] algorithm. Cells maintained in the in vitro culture for the same period were used as controls. At least 1000× library coverage was maintained during every step of the experiments, including cell culture, in vivo screening, and sequencing.

### 4.4 CRISPR Gene Editing

ITGB1 knockdown cells were generated with the CRISPR/Cas9 system. Specifically, primer pairs that contained sequences for either control sgRNAs or sgRNAs targeting either mouse or human ITGB1 [120, 121] were designed and subsequently cloned into the all‐in‐one LentiCRISPR V2 vector (Addgene, #52961, #83480) [121]. Viral particles were produced by transfecting HEK293T cells with the LentiCRISPR V2 vector alongside the packaging plasmid. Post‐transfection, the generated lentiviruses were centrifuged, passed through a filter to remove cell debris, and preserved at −80°C. For infection experiments, MDA‐MB‐231A or 4T1 cells were exposed to the respective viruses for a duration of 48 h in the presence of 8 µg/mL polybrene. Following virus removal, the cells underwent selection using 2 µg/mL of puromycin or 5 µg/mL of blasticidin.

The following sgRNA sequences were used [121]:

Control (LacZ, for human and mouse cells): UUGGGAAGGGCGAUCGGUGC

Control (non‐targeting, for human cells): GAACGUAGAAAUUCCCAUUU

Control (non‐targeting, for mouse cells): AGUCCGGUCGAAAUCUGUAU

*ITGB1* (for human cells): UGCUGUGUGUUUGCUCAAAC

*ITGB1* (for human cells): GAACGGGGUGAAUGGAACAG

*Itgb1* (for mouse cells): AAUGUCACCAAUCGCAGCAA

*Itgb1* (for mouse cells): GUGCUUAGUCUUACUGACAG

*ITGA2* (for human cells): AAUAACUUACGCUGAGUUGC

*ITGA3* (for human cells): CGGGAGCUCGCUGUGCCCGA

*ITGA5* (for human cells): GGGGCAACAGUUCGAGCCCA

*ITGA6* (for human cells): UCAAAUUCAAUCCGUGUACA

*Itga2* (for mouse cells): CAGCAGCUUACGAACCCACA

*Itga3* (for mouse cells): ACUUGCCCACCAUGCGCCGC

*Itga5* (for mouse cells): UCCCCAAGGCUCCCGGAUUC

*Itga6* (for mouse cells): UCAAAUUCAAUCCGUGUACA

### 4.5 Analysis of Clinical Dataset

Publicly available TNBC datasets for mRNA expression and survival analysis were obtained from Gene Expression Omnibus (GSE76250, GSE45827, and GSE202776). Data analysis as implemented in the R package. To estimate overall survival (OS), Kaplan‐Meier survival curves are generated using the survfit function, followed by a log‐rank test to assess differences in OS between the high‐ and low‐risk groups. Correlation of ITGB1 expression with TAM cell markers across the TCGA BRCA (GDC) mRNA dataset and the CPTAC mRNA dataset.

### 4.6 Mouse Tumor Model for Therapeutic Efficacy

In the mammary fat pad inoculation model, different cell lines were used for various experimental conditions. MDA‐MB‐231A cells (5 × 10^4^) or 4T1 cells (1 × 10^4^) were used for the mammary fat pad injection. For the *ITGB1*‐KO model, Ctrl‐KO and *ITGB1*‐KO MDA‐MB‐231A or 4T1 cells were used. For the *ITGA*‐KO model, MDA‐MB‐231A (Ctrl‐KO, *ITGB1*‐KO, *ITGA2*‐KO, *ITGA3*‐KO, *ITGA5*‐KO or *ITGA6*‐KO) cells and 4T1 (Ctrl‐KO, *Itgb1*‐KO, *Itga2*‐KO, *Itga3*‐KO, *Itga5*‐KD, *Itga6*‐KD, *Itga2*/*Itgb1*‐KO, *Itga5*/*Itgb1*‐KO or *Itga6*/*Itgb1*‐KO) cells were used. For the rescue model, *Itgb1*‐KO 4T1 expressing rescue‐*ITGB1*‐(ΔICD, Δ1‐380, ΔICD.1‐380, R1/2/3/4, R1, R2, R3, R4, R1/3, R1/4, R3/4 or R1/3/4) or rescue‐*ITGB5* were injected. For the scFv antibody model, MDA‐MB‐231A (scFv‐M200, or scFv‐OS2966) cells (5×10^5^) were used. These cells were suspended in RPMI medium supplemented with 15% Matrigel Matrix (Corning) and were then injected into the mammary fat pads of 8‐ to 12‐week‐old female Rag2^−/−^, γc^−/−,^ or BALB/c mice. Tumor progression and metastasis were monitored using bioluminescent imaging, and tumor volumes were measured.

In the B6H12 treatment model, Ctrl‐KO and *ITGB1*‐KO MDA‐MB‐231A cells were injected into the mammary fat pads of 8‐ to 12‐week‐old female Rag2^−/−^, γc^−/−^ mice. Before treatment, mice were randomized. Starting 7 days after tumor inoculation, mice were treated once per week with either PBS or B6H12 antibodies (50 µg per mouse) via the retro‐orbital route. Tumor volumes were monitored.

In the OS2966 treatment model, MDA‐MB‐231A cells were suspended in RPMI medium with 15% Matrigel Matrix and injected into the mammary fat pads of 8‐ to 12‐week‐old female Rag2^−/−^, γc^−/−^ mice. Before treatment, mice were randomized. Mice received OS2966 antibody (50 µg per mouse) or PBS twice per week via the intratumoral or retro‐orbital route, starting either 7 or 21 days after tumor inoculation. Tumor volumes were monitored.

For the intravenous (IV) inoculation model, Ctrl‐KO 4T1 (5 × 10^5^) and *Itgb1*‐KO 4T1 (5 × 10^5^) cells were suspended in RPMI medium. These cells were then IV injected into 8‐ to 12‐week‐old female BALB/c mice. Bioluminescent imaging was utilized to monitor tumor progression and metastasis.

For the rechallenge model, *Itgb1*‐KO 4T1 cells were suspended in RPMI medium supplemented with 15% Matrigel Matrix and then injected into the mammary fat pads of 8‐ to 12‐week‐old female BALB/c mice. After 42 days, Ctrl‐KO 4T1 cells were reintroduced to the contralateral sites of naïve mice or those primed with initial *Itgb1*‐KO 4T1 tumor engraftment. Tumor volumes were monitored.

In the T cell depletion model, 4T1 cells and *Itgb1*‐KO 4T1 cells were injected into the mammary fat pads of 8‐ to 12‐week‐old female BALB/c mice. Mice were treated with either vehicle, CD4‐ or CD8‐neutralizing antibodies (200 µg per mouse) via intraperitoneal (IP) route twice per week, starting from three days prior to tumor inoculation. Tumor volumes were monitored.

In the double knockout model, Ctrl‐KO and *Cd274*‐KO 4T1‐Ctrl‐KO or *Itgb1*‐KO cells (5 × 10^5^) were suspended in RPMI medium supplemented with 15% Matrigel Matrix and then injected into the mammary fat pads of 8‐ to 12‐week‐old female BALB/c mice. Tumor volumes were monitored.

Bioluminescent imaging was employed to monitor tumor progression and metastasis, as described previously [21, 84]. Specifically, tumor‐bearing mice were intraperitoneally injected with D‐luciferin (Syd Labs) dissolved in PBS, at a dosage of 140 mg luciferin/kg body weight. Imaging sessions were conducted using the Lago X system (Spectral Instruments Imaging), and bioluminescence signals were analyzed with the Aura Image software, also from Spectral Instruments Imaging.

Tumor dimensions were recorded by measuring their length (L) and width (W). The tumor volumes were then calculated using the formula (L×W×W)/2. We ensured that the maximal tumor size did not exceed 15 mm in diameter, in accordance with our approved animal protocol.

In experiments in which tumors grew to a predefined size prior to intervention, mice were randomized into treatment groups before administration of any therapeutic agents. Randomization was performed to ensure comparable tumor burden across groups at the initiation of treatment. In all experiments, investigators responsible for data acquisition and outcome assessment were blinded to group allocation and experimental design to minimize bias.

### 4.7 Mouse Tumor Model for Flow Cytometry Phenotyping

Ctrl‐KO, *ITGB1*‐KO MDA‐MB‐231A, or 4T1 cells, as well as *Itgb1*‐KO 4T1 cells expressing Rescue‐*ITGB1* (ΔICD, ΔICD.ETD, R1), were injected into the mammary fat pads of Rag2^−/−^, γc^−/−^ mice or BALB/c mice. In one model using MDA‐MB‐231A cells, tumors were additionally treated with either PBS or OS2966. Upon euthanasia of the mice, tumor tissues were carefully extracted and chopped into approximately 1 mm fragments. For digestion, the tissue was treated with plain RPMI medium supplemented with 2 mg/mL Collagenase I, 2 mg/mL Hyaluronidase, and 25 µg/mL DNase I. Specifically, 3 mL of this digestion medium was applied to every 0.2 gram of tumor tissue. This tissue mixture was then digested at 37°C with vigorous shaking for 45 min. The digestion was quenched and passed through a 60‐micron mesh. After washing twice with PBS, the samples were subjected to flow cytometry antibody staining.

### 4.8 Flow Cytometry

Flow cytometry analysis was acquired using the BD LSRFortessa X‐20 or Cytek Aurora, while cell sorting was performed on the BD FACSAria III flow cytometers. The study employed the following fluorescent dye‐labeled antibodies, all acquired from BioLegend: mCD45 (30‐F11), mCD4 (GK1.5), mCD8 (53‐6.7), mCD44 (IM7), mCD62L (MEL‐14), mCD11b (M1/70), mCD11c (N418), mF4/80 (BM8), mGr‐1 (RB6‐8C5), mLy‐6C (HK1.4), mCD206 (C068C2), mCD3 (17A2), hITGB1 (TS2/16), and mITGB1 (HMβ1‐1), hITGB5 (AST‐3T), hITGA2 (P1E6‐C5), hITGA3 (ASC‐1), hITGA5 (NK1‐SAM‐1), h/mITGA6 (GoH3), mITGA2 (HMa2), mITGA3 (CD49c), mITGA5 (MFR5), HA.11 (poly9023), hIgG Fc (QA19A42), mTREM2 (6E9), mCD204 (1F8C33), mCLEC4D (3A4), mFCGR4 (9E9), mNOS2 (W16030C) and mNK1.1 (S17016d).

### 4.9 Western Blotting

Proteins extracted from cell fractionation or cells lysed in radioimmunoprecipitation assay (RIPA) buffer were quantified using the bicinchoninic acid (BCA) protein assay. These protein samples were mixed with a loading buffer containing 62.5 mM Tris‐HCl (pH 6.8), 10% glycerol, 2% SDS, 0.01% bromophenol blue, and 100 mM dithiothreitol, followed by separation via SDS‐polyacrylamide gel electrophoresis (SDS‐PAGE). For immunoblotting, we used the HRP anti‐human IgG (from BioLegend) antibody.

### 4.10 Bulk‐RNA Sequencing

Tumor cells were harvested either from tumor tissues or directly from in vitro cultures. Total RNA was subsequently extracted using a Qiagen RNA extraction kit and prepped for sequencing. RNA samples from three individual mice were processed as triplicates and sent to Novogene for RNA sequencing, conducted on the Illumina NovaSeq 6000 platform. RNA‐seq raw data were processed using Partek Flow. In brief, the sequencing reads were aligned to the human genome utilizing the Spliced Transcripts Alignment to a Reference (STAR 2.5.3a) tool. These aligned reads were subsequently quantified to the transcriptome, referencing hg38 for humans (Ensembl Transcripts release 109). The data then underwent Median Ratio normalization. For differential gene expression analysis, we employed DESeq2. Hierarchical clustering was achieved using the heatmap function within RStudio. Differentially expressed genes (with a fold change greater than 2 and a *p*‐value less than 10e‐5) were visualized in a volcano plot.

### 4.11 3D culture

The 96‐well plates were precoated with Matrigel as a basement membrane by adding 100 µL of 50% Matrigel to each well, followed by incubation at 37°C for 15 min. MDA‐MB‐231A or 4T1 (4000 cells/well) cells were suspended in a medium containing 2% Matrigel and then seeded directly onto the Matrigel‐precoated wells. Fluorescence microscopy was used to obtain images. The wells were subsequently treated with plain RPMI medium supplemented with 2 mg/mL Collagenase I and 2 mg/mL Hyaluronidase, and the cells were collected and quantified.

### 4.12 Cell Proliferation and Wound‐Healing Assay

MDA‐MB‐231A or 4T1 cells (2,500 cells/well), lentivirally stable‐transduced to express the enhanced GFP (eGFP)‐luciferase fusion protein, were seeded in 96‐well plates, with each well containing a total volume of 150 µL. Cell proliferation was assessed on days 1, 2, 3, 4, and 5 by measuring luminescence signals with a Cytation 3 imaging reader (BioTek). The proliferation rates for each well were determined by normalizing the luminescence values against their respective baseline readings from day 1.

MDA‐MB‐231 or MDA‐MB‐231A cells (2 × 10^5^ cells/well) were seeded in each well of a 12‐well plate. After the cells adhere to the bottom of the plate, use a tip to draw a line on the bottom of the plate, simulating a wound. After 24 h, observe the wound healing situation.

### 4.13 Cell Viability Assay

Tumor cells at 70% confluency culture were dissociated and stained with Annexin‐V‐PE following the manufacturer's protocol, then stained with Sytox‐Blue before being subjected to flow cytometry analysis for viability/apoptosis assessment.

### 4.14 Single‐Cell RNA Sequencing

Ctrl‐KO, *Itgb1*‐KO, or *ITGB1*‐rescue 4T1 cells were inoculated into the mammary fat pads of Rag2^−/−^, γc^−/−^ mice. Upon euthanasia of the mice, tumor tissues were carefully extracted and chopped into approximately 1 mm fragments. The tissue was treated with plain RPMI medium supplemented with 2 mg/mL Collagenase I, 2 mg/mL Hyaluronidase, and 25 µg/mL DNase I, and digested at 37°C with vigorous shaking for 45 min. The digestion was quenched and passed through a 60‐micron mesh. After washing twice with PBS, the single cell suspension samples were stained with Live/Dead dye, anti‐mouse CD45, and anti‐mouse CD11b antibodies. Live CD45^+^CD11b^+^ populations were sorted out for single‐cell library generation.

Cells were captured using a Chromium controller (10×Genomics) with a single‐cell 3’ Reagent kit V3.1 (10× Genomics, PN‐1000121) targeting ∼5000–7000 cells per sample. Single‐cell RNA‐seq libraries were prepared following the manufacturer's instructions. The quality of cDNA and sequencing libraries was assessed using a High Sensitivity DNA Chip (Agilent Technologies, #5067‐4626). Library concentration was determined using a Qubit High Sensitivity DNA assay Kit (ThermoFisher Scientific). Subsequently, the libraries were sequenced on an Illumina NovaSeq 6000 platform (Illumina) at The Translational Genomics Research Institute (TGen), with a sequencing depth of 50–100K reads per cell.

Single‐cell RNA‐seq reads were quantified by Cell Ranger‐7.1.0 and aligned to the Mus_musculus. GRCm39.105 transcriptome reference. Subsequently, the raw data from different conditions were integrated and subjected to filtering, which excluded cells expressing fewer than 500 genes or more than 6000 genes or harboring more than 5% mitochondrial reads. The processed data were then subjected to normalization and log‐transformed employing the Normalize Data function within Seurat (v4.3.0). Among the initial cohort of 11 720 sequenced cells, a total of 9517 cells successfully passed the quality control measures, collectively expressing 20 654 genes. The Seurat vst algorithm was applied to identify the top 2000 highly variable genes. Subsequently, the data were scaled, and a jackstraw analysis was executed. The determination of principal components (PCs) for downstream analysis was guided by an elbow plot of standard deviation in each PC, which revealed that the first 15 PCs captured the predominant proportion of the true signals. These selected PCs were employed to generate the K‐nearest neighbor (KNN) neighborhood graph. Clustering was conducted utilizing the Louvain algorithm with a resolution parameter of 0.8 and visualized in UMAP‐reduced dimensions. Differentially expressed genes within each cluster were calculated using the Wilcox analysis.

### 4.15 Immunohistochemistry

Tissue samples were fixed in 10% formalin and forwarded to the Pathology of the City of Hope Comprehensive Cancer Center for H&E and IHC staining. Specifically, the tumor was embedded in paraffin wax to permit sectioning. For immunohistochemistry, antigen retrieval was performed under high pressure and temperature in 0.01 nmol/L citrate buffer, followed by treatment with 3% H_2_O_2_ to block the endogenous peroxidase activity. Sections were treated with 3% bovine serum albumin to block non‐specific binding and then incubated with CD3 antibodies overnight at 4°C. Secondary antibodies were used for 1 h at 37°C. Image acquisition was performed using the Aperio ScanScope from Leica Biosystems. Visualization was facilitated by NDP.view2, and image analysis was carried out.

### 4.16 Protein Expression and Purification

Human truncated α5 (1‐664), β1 (1‐465), and the light chain and heavy chain of OS2966 were cloned into the pEG BacMam vector (Thermo Fisher Scientific), respectively, for protein expression in HEK293 cells (ExpressionSystems) using the BacMam system [122]. The α5 was fused with a C‐terminal strep‐tag linked with a TEV site, while the β1 was fused with a C‐terminal his‐tag. Additionally, an acidic and a basic coiled‐coil segment with a cysteine residue were fused to the C‐termini of α5 and β1, respectively, to facilitate coiled‐coil clasp formation [123]. The α5 and β1 and the light chain and heavy chain of OS2966 were co‐expressed in HEK293 cells separately as secreted proteins.

The soluble integrin headpiece α5β1 complex was purified from cell culture supernatant using Strep‐Tactin resin (IBA Lifesciences) and further purified by size exclusion chromatography using a Superdex 200 Increase 100/300 GL column (GE Healthcare). The purified α5β1 complex was concentrated and stored at −80°C until use. OS2966 was purified from cell supernatant using Protein A resin (GenScript) and further digested the Fc fragment by Ficin resin (Thermo Fisher Scientific) to produce the Fab fragment.

Purified α5β1 complex and OS2966 Fab fragment were mixed at a molar ratio of 1:1 at 4°C overnight. The complex was purified by size exclusion chromatography on a Superdex S200 increase column in a buffer composed of 20 mM HEPES pH 7.5, and 100 mM NaCl. The complex fractions were collected and concentrated to 0.1 mg/mL for making cryo‐EM grids.

### 4.17 Cryo‐EM Sample Preparation and Data Acquisition

For cryo‐EM grid preparation, 3 µL of purified complex was applied to glow‐discharged holey carbon grids (Quantifoil, Cu 300 R1.2/1.3). Grids were plunge‐frozen in liquid ethane using Vitrobot Mark IV (Thermo Fischer Scientific). Cryo‐EM data were collected using a Titan Krios transmission electron microscope, equipped with a Falcon 4 Summit direct electron detector and an energy filter. A total of 11 386 movies were collected. Data acquisition was performed with EPU software, with a physical pixel size of 0.72 Å and a total electron dose of 50 e^−^/Å^2^.

### 4.18 Data Processing, 3D Reconstruction, and Modeling Building

Image stacks were subjected to patch motion correction using cryoSPARC [124]. The contrast transfer function (CTF) parameters were calculated using the patch CTF estimation tool in cryoSPARC. A total of 8 195 861 particles were autopicked and then subjected to 2D classification. After ab initio reconstruction and heterogeneous refinement, 2 013 523 particles were subjected to nonuniform refinement to generate a map with an indicated global resolution of 2.56 Å at a Fourier shell correlation (FSC) of 0.143.

The Alphafold‐predicted structures of α5, β1, and OS2966Fab served as initial models for model rebuilding. The model was initially docked into the EM density map using Chimera [125], followed by iterative manual building in COOT [126] and refinement in Phenix [127]. The final model statistics were validated by MolProbity [128]. Chimera and PyMOL (https://pymol.org/2/) were used to prepare structural figures.

### 4.19 Software

Mouse BLI quantification and imaging were executed using both Aura Imaging software (Spectral Instruments Imaging) and Living Image Software (IVIS Imaging Systems). Schematic representations were crafted with BioRender (Biorender.com). Schematic illustrations were created in BioRender. Song, N. (2026) https://BioRender.com/tqre53j. Flow cytometry data analysis was done using FlowJo V9.0 (TreeStar). Statistical assessments and main data visualization were achieved using GraphPad Prism (Version 9.5.0 (525)). Immunohistochemistry images were acquired via the Aperio ScanScope from Leica Biosystems, viewed with NDP.view2, and analyzed using ImageJ (version 1.53t). For RNA sequencing, bulk data were analyzed with Partek Flow (Partek) and visualized in RStudio, while single‐cell data analyses were conducted with CellRanger and Seurat.

### 4.20 Statistics

All data are expressed as the mean ± SD. Statistical significance was tested by two‐tailed *t*‐tests, one‐way ANOVA with multiple comparison tests, or two‐way ANOVA with multiple comparison tests. For animal experiments with repeated measurement (BLI or tumor volume), data were analyzed with RM two‐way ANOVA with Tukey's multiple comparison test. Statistical analyses were achieved by GraphPad Prism (Version 9.5.0 (525)). All comparisons are 2‐tailed. In all cases, *P* < 0.05 was considered statistically significant.

### 4.21 Study Approval

All procedures involving animals were duly approved by the Administrative Panel on Laboratory Animal Care at the City of Hope Comprehensive Cancer Center.

## Author Contributions

N.S., S.C., L.W., C.Z., and M.F. designed the experiments. N.S., S.C., L.W., J.D., X.C., S.S., J.W., and M.F. performed the experiments. N.S., S.C., L.W., L.Y., Y.W., C.‐D.C., C.Z., and M.F. analyzed the data. S.T.R., Y.W., C.‐D.C., C.Z., and M.F. provided scientific input. N.S. and S.C. prepared the figures. N.S., S.C., C.Z., and M.F. wrote the manuscript. All authors edited the manuscript. M.F. and C.Z. conceived and supervised the study.

## Funding

The authors thank the excellent technical support of Core Facilities at City of Hope, including the Analytical Cytometry, Animal Resource Center, Small Animal Imaging, Pathology, and Integrative Genomics, supported by the NIH P30CA033572. The authors thank Feng and Zhang lab members for their assistance and input in this work. This work was supported by the NIH grants K99/R00CA201075 (MF), R01CA255250 (MF), R01CA258778 (MF), R21CA280317 (MF), R35GM128641 (CZ), R01CA233691 (C‐DC), and R01CA278050 (C‐DC); the CIRM training program EDUC4‐12772 (NS); the V Foundation for Cancer Research V Scholar Award V2018‐012 (MF); and the startup research funding from City of Hope (MF). Funds from anonymous donors helped accelerate this study.

## Conflicts of Interest

The authors declare no conflicts of interest.

## Supporting information




**Supporting File 1**: advs74151‐sup‐0001‐FigureS1‐S11.docx.

**Supporting File 2**: advs74151‐sup‐0002‐TableS1.xlsx.

**Supporting File 3**: advs74151‐sup‐0003‐TableS2.xlsx.

**Supporting File 4**: advs74151‐sup‐0004‐TableS3.xlsx.

## Data Availability

Bulk RNA‐seq data have been deposited in Gene Expression Omnibus (GEO) with the number GSE244508. The 3D cryo‐EM density map of the integrin α5β1 headpiece with the Fab fragment of OS2966 has been deposited in the Electron Microscopy Data Bank under the accession number EMD‐49184. Atomic coordinates for the atomic model have been deposited in the Protein Data Bank (PDB) under the accession number 9NAB. All other data supporting the findings of this study are available from the corresponding author upon request.
