# Supplementary material for: ITGB1 Regulates Triple‐Negative Breast Cancer Development by Modulating the Tumor Microenvironment
Source: Adv Sci (Weinh). 2026 Feb 3;13(20):e13672. doi: 10.1002/advs.202513672 (PMC13067782; doi:10.1002/advs.202513672)
Supplement: Supplementary file 1 — Supporting File 1: advs74151‐sup‐0001‐FigureS1‐S11.docx. [file ADVS-13-e13672-s002.docx]

**Supplemental Figures 1-11**

**ITGB1 Regulates Triple-Negative Breast Cancer Development by Modulating the Tumor Microenvironment**

Nuozi Song^1†^, Siqi Chen^1†^, Lei Wang^2†^, Jessica Dang^1‡^, Xu Cao^1‡^, Stephanie Singh^2^, Lu Yang^3,4^, Jinhui Wang^5^, Steven T. Rosen^6,7,8^, Yingyu Wang^6^, Chun-Wei D. Chen^3,4,9^, Cheng Zhang^2*^, Mingye Feng^1^**^*^**

***** Correspondence: Cheng Zhang, [chengzh@pitt.edu](mailto:chengzh@pitt.edu), Mingye Feng, [mfeng@coh.org](mailto:mfeng@coh.org)

**
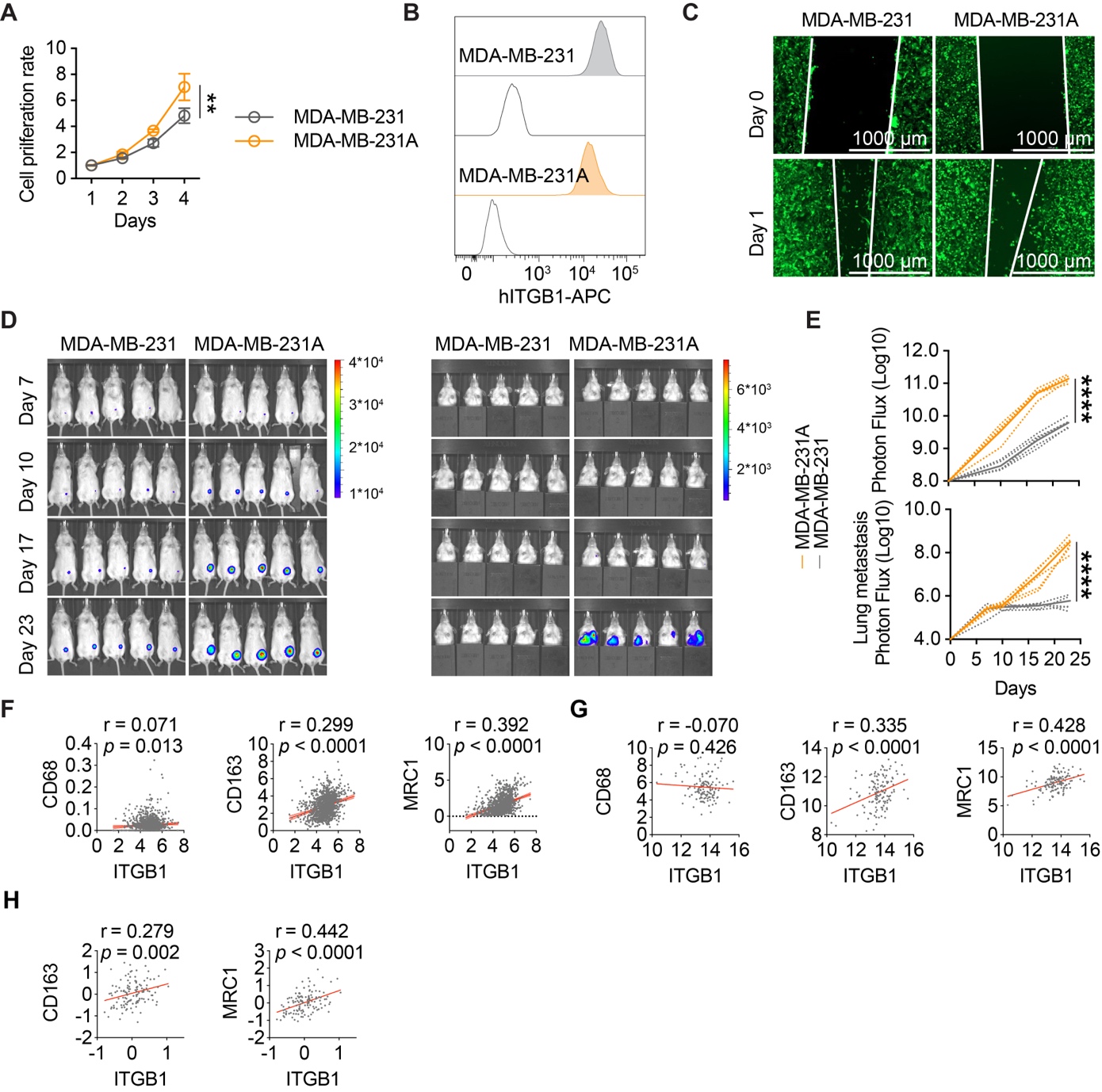
**

**Figure S1. Identification of ITGB1 as a critical regulator of breast tumorigenesis.**

(**A**) Examination of the *in vitro* proliferation of MDA-MB-231 and MDA-MB-231A cells. *n* = 3 mice; two-way RM ANOVA. (**B**) FACS plots showing the expression of ITGB1 on MDA-MB-231 and MDA-MB-231A. (**C**) Representative wound-healing images of MDA-MB-231 and MDA-MB-231A lines at Day 0 and Day 1. (**D-E**) Bioluminescence imaging (D) and quantification (E) of tumor growth (left) and the metastasis (right) in Rag2^-/-^, γc^-/-^ mice inoculated with MDA-MB-231 or MDA-MB-231A cells. *n* = 5 mice; two-way RM ANOVA. (**F-G**) Scatter plots showing correlations between log2 *ITGB1* mRNA levels and macrophage (CD68, CD163, MRC1) markers using the TCGA BRCA (GDC) mRNA dataset (F) and the CPTAC mRNA dataset (G). (**H**) Scatter plots showing correlations between ITGB1 and CD163/MRC1 (log2 relative protein abundance) using the CPTAC proteomics dataset. In all figures, ns, no significance, ****P* < 0.001, *****P* < 0.0001.

**
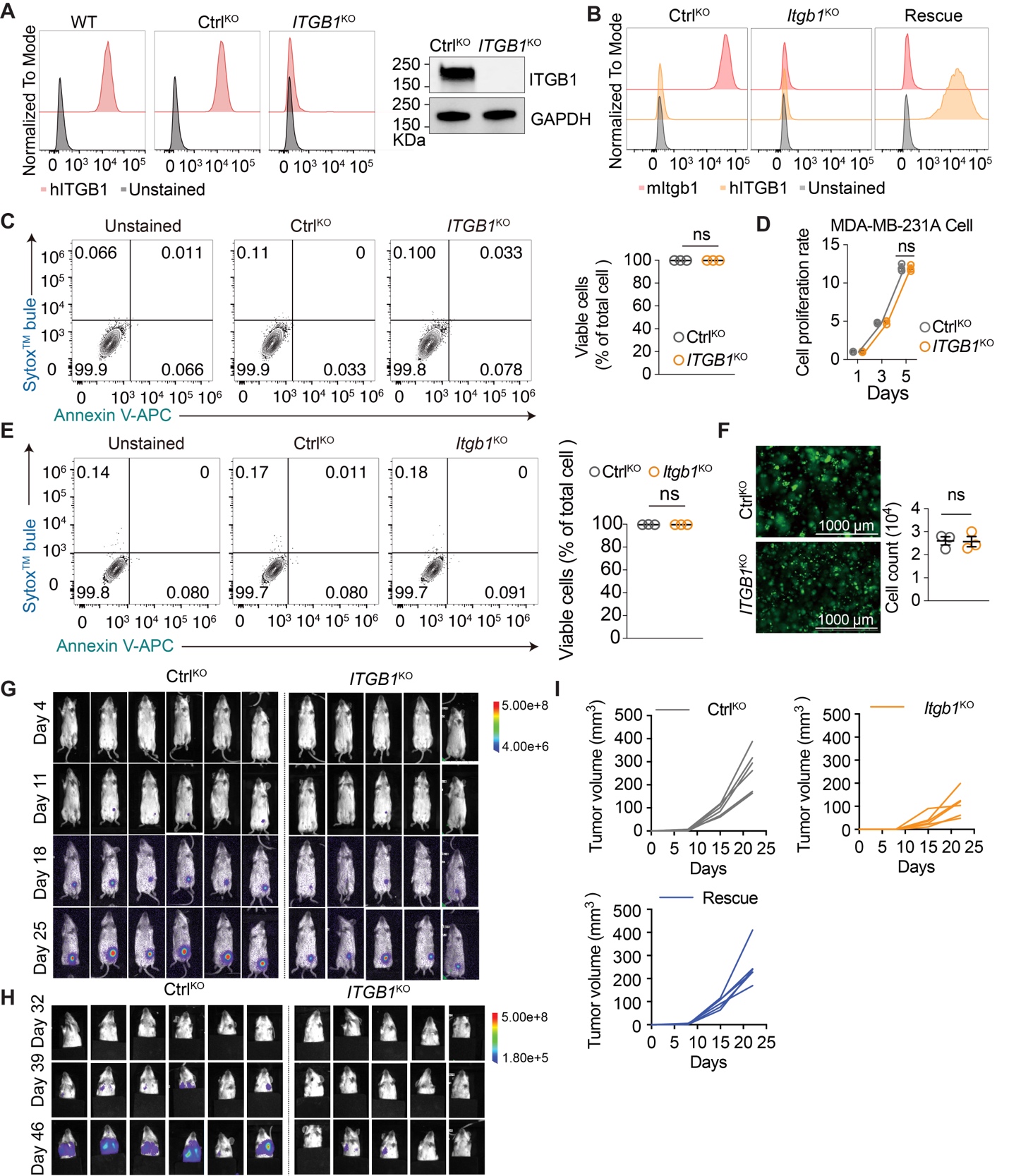
**

**Figure S2. The expression and viability of Ctrl-KO or *ITGB1*-KO MDA-MB-231A and 4T1 cells.**

(**A**) FACS plots (left) and immunoblot analysis (right) showing the expression of ITGB1 on WT, Ctrl-KO, or *ITGB1*-KO MDA-MB-231A cells. (**B**) FACS plots showing the expression of human or mouse ITGB1 on Ctrl-KO, *ITGB1*-KO, and *ITGB1*-Rescue (*ITGB1*-KO with re-introduced hITGB1) 4T1 cells. (**C**) Examination of the viability of Ctrl-KO or *ITGB1*-KO MDA-MB-231A cells, by Annexin V and Sytox blue staining. Representative FACS plots (left) and quantification of viable cells (right) are shown. *n* = 3; unpaired *t*-test. (**D**) Examination of the *in vitro* proliferation of Ctrl-KO or *ITGB1*-KO MDA-MB-231A cells. *n* = 3; two-way RM ANOVA. (**E**) Examination of the viability of Ctrl-KO or *ITGB1*-KO 4T1 cells, by Annexin V and Sytox blue staining. Representative FACS plots (left) and quantification of viable cells (right) are shown. *n* = 3; unpaired *t*-test. (**F**) Spheroid of MDA-MB-231A cells at day 7 post-embedding in a 3D Matrigel. Representative photomicrographs (left) and quantification (right) of cell counts are shown. (**G-H**) Bioluminescence imaging showing tumor growth (G) and lung metastasis (H) in MDA-MB-231A-engrafted Rag2^-/-^, γc^-/-^ mouse. *n* = 6 (Ctrl-KO) or 5 (*ITGB1*-KO) mice. (**I**) Tumor growth curve of individual mice in Figure 1J. *n* = 6 mice per group. In all figures, ns, no significance; Data are represented as mean ± SD.

**
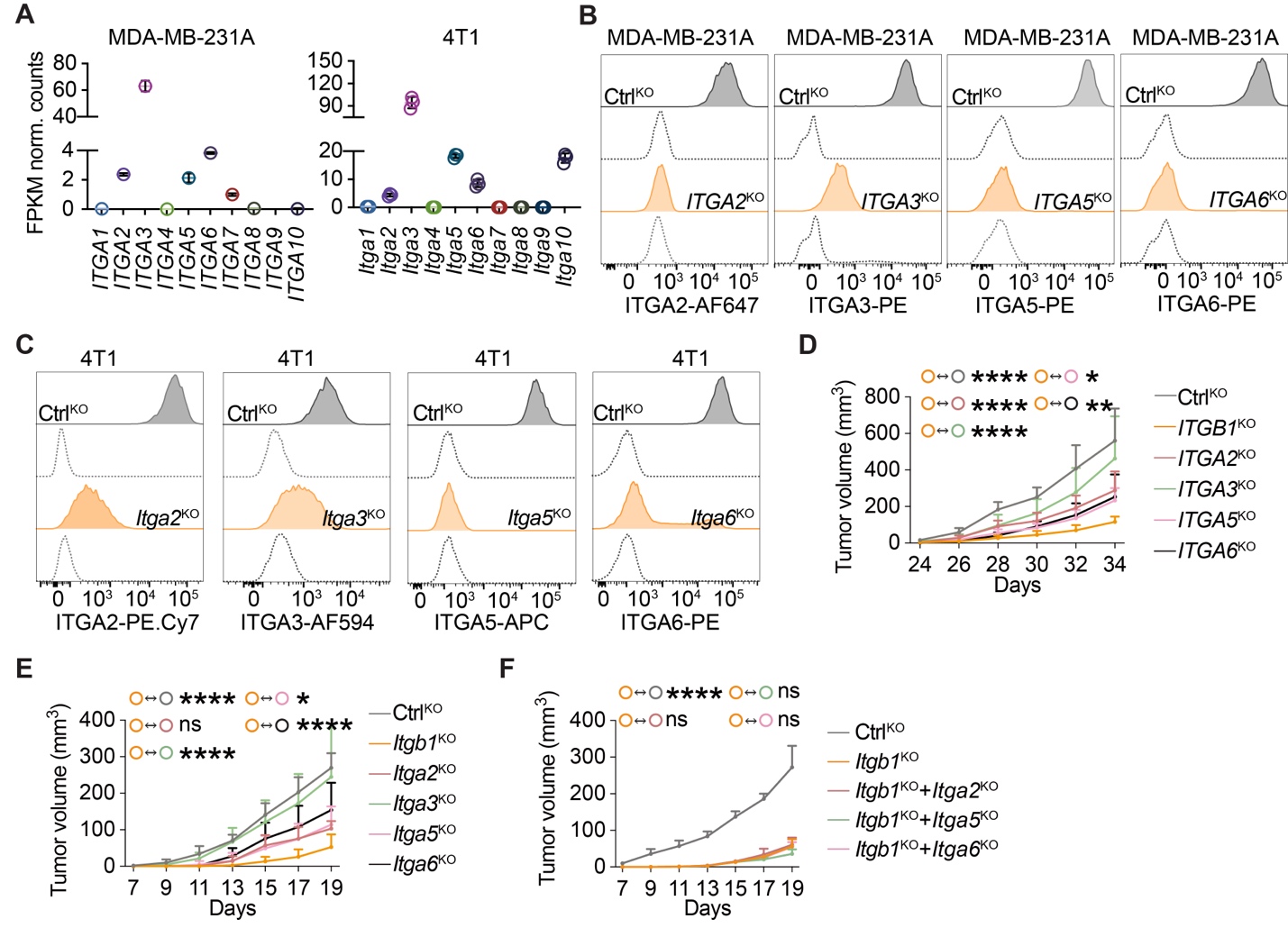
**

**Figure S3. The expression of ITGAs in the MDA-MB-231A and 4T1 cells.**

(**A**) The gene count of *ITGA* in MDA-MB-231A and 4T1 tumor cells by bulk-RNA sequencing. *n* = 3. (**B**) FACS plots showing the expression of ITGA2, ITGA3, ITGA5, and ITGA6 in Ctrl-KO, *ITGA2*-KO, *ITGA3*-KO, *ITGA5*-KO, or *ITGA6*-KO MDA-MB-231A cells. (**C**) FACS plots showing the expression of ITGA2, ITGA3, ITGA5, or ITGA6 in Ctrl-KO, *Itga2*-KO, *Itga3*-KO, *Itga5*-KO, and *Itga6*-KO 4T1 cells. (**D-E**) Growth of tumors developed by Ctrl-KO, *ITGB1*-KO, *ITGA2*-KO, *ITGA3*-KO, *ITGA5*-KO, or *ITGA6*-KO MDA-MB-231A (D) or 4T1 (E) cells in Rag2^-/-^, γc^-/-^ mice. *n* = 6 mice; two-way RM ANOVA with multiple comparisons at Day34 (D) and Day19 (E). (**F**) Growth of tumors developed by Ctrl-KO, *Itgb1*-KO or double KO of *Itgb1*/*Itga2*, *Itgb1*/*Itga5*, *Itgb1*/*Itga6* 4T1 cells in Rag2^-/-^, γc^-/-^ mice. *n* = 4 mice; two-way RM ANOVA with multiple comparison test. ns, no significance, **P* < 0.05, ****P* < 0.001, *****P* < 0.0001. Data are represented as mean ± SD.

**
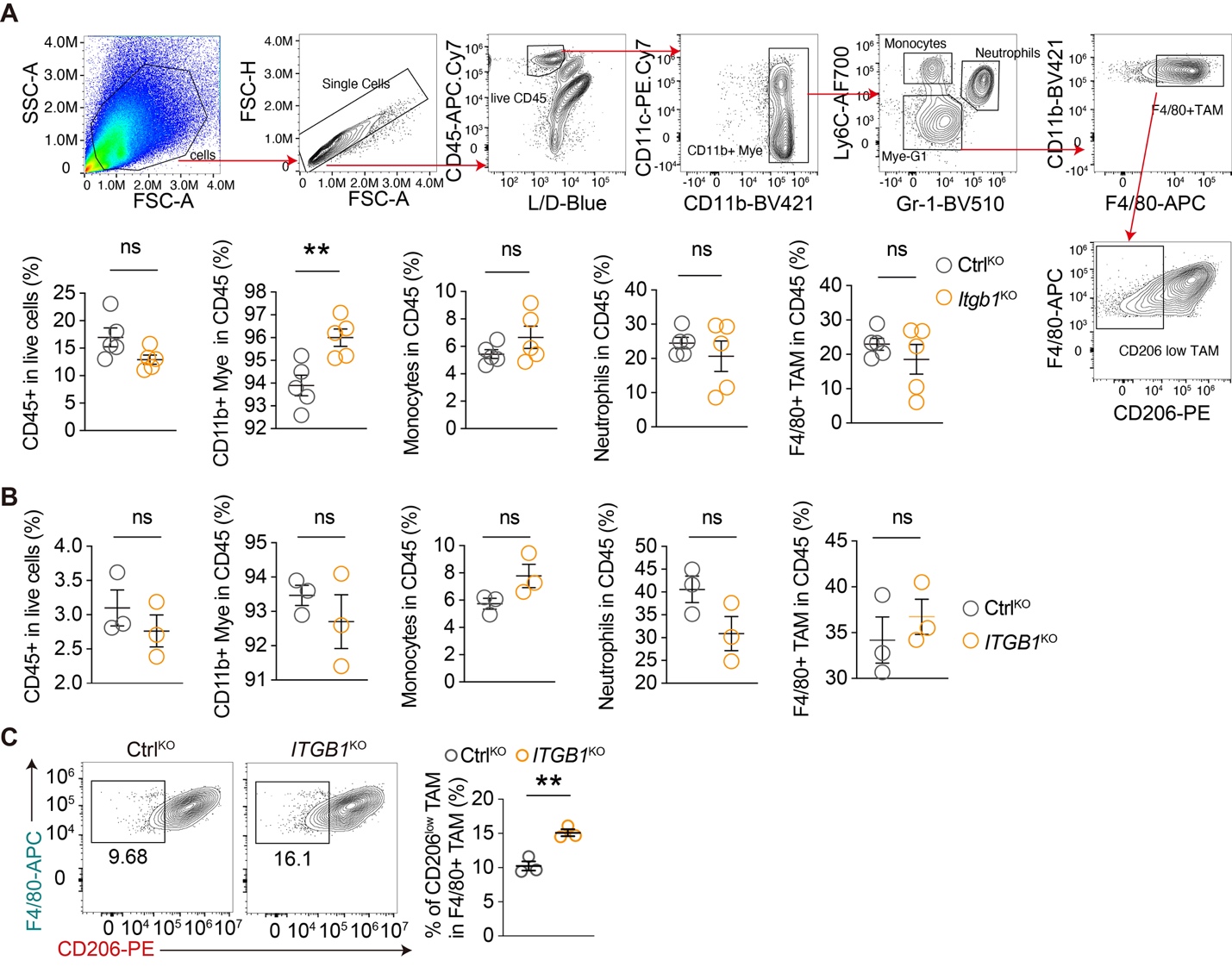
**

**Figure S4. Flow cytometry phenotyping of 4T1 and MDA-MB-231A tumor tissues.**

(**A**) FACS gating strategy and quantification showing the percentage of immune cells (CD45^+^), CD11b^+^ myeloid cells (CD45^+^CD11b^+^), monocytes (CD45^+^CD11b^+^Ly6c^+^Gr-1^low^), neutrophils (CD45^+^CD11b^+^Ly6c^+^Gr-1^high^ and F4/80^+^TAM (CD45^+^CD11b^+^Ly6c^-^Gr-1^low^F4/80^+^) in mouse tumor tissues from Ctrl-KO or *Itgb1*-KO 4T1-engrafted Rag2^-/-^, γc^-/-^ mice. *n* = 5 mice; unpaired *t*-test. (**B-C**) FACS quantification showing the percentage of immune cells (CD45^+^), CD11b^+^ myeloid cells (CD45^+^CD11b^+^), monocytes (CD45^+^CD11b^+^Ly6c^+^Gr-1^low^), neutrophils (CD45^+^CD11b^+^Ly6c^+^Gr-1^high^), F4/80^+^TAM (CD45^+^CD11b^+^Ly6c^-^Gr-1^low^F4/80^+^) (B) and CD206-low F4/80^+^ TAMs among total TAMs (CD45^+^CD11b^+^Ly6c^-^Gr-1^low^F4/80^+^) (C) in mouse tumor tissues from Ctrl-KO or *ITGB1*-KO MDA-MB-231A-engrafted Rag2^-/-^, γc^-/-^ mice. *n* = 3 mice; unpaired *t*-test. In all figures, ns, no significance, ***P* < 0.01; Data are represented as mean ± SD.

**
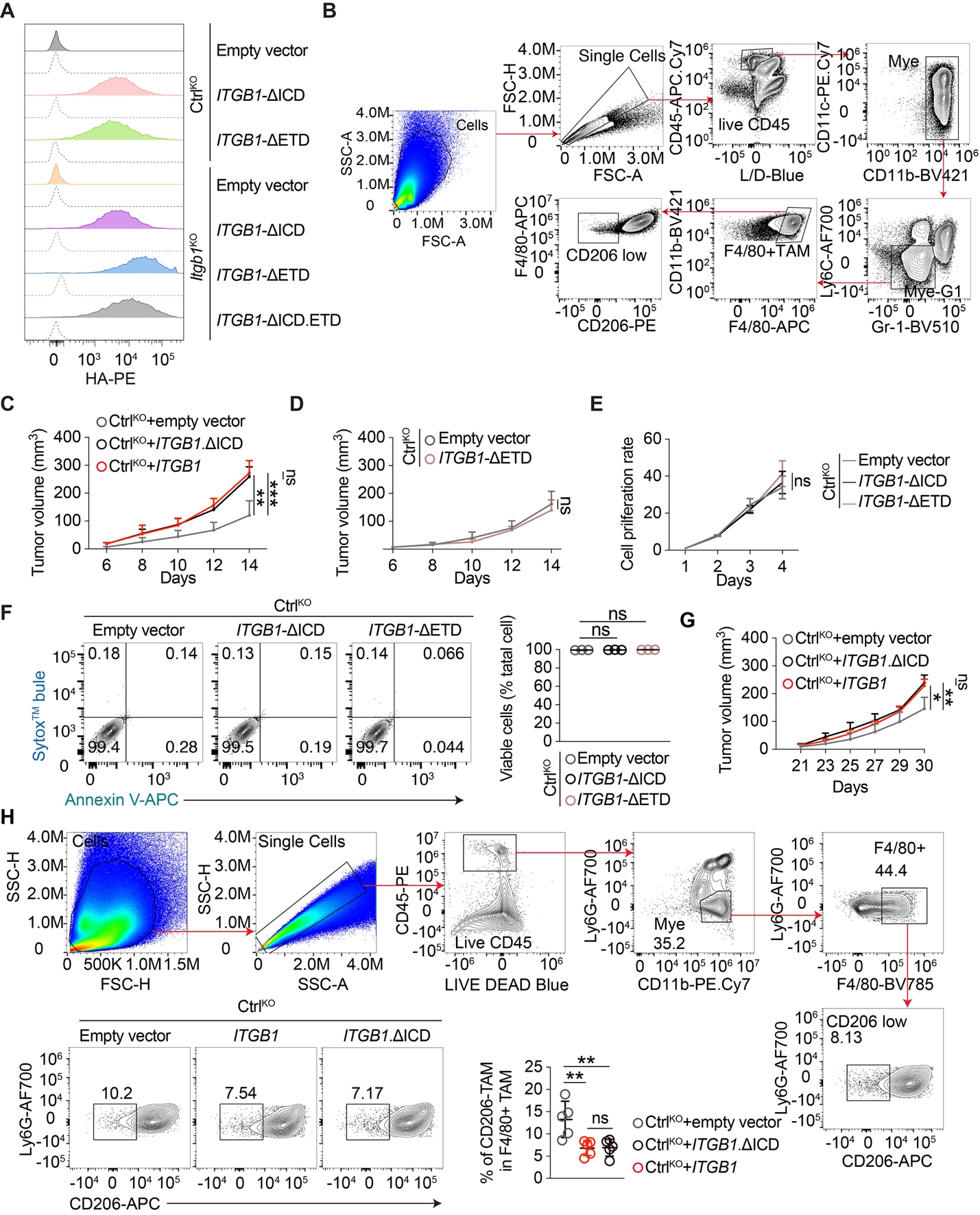
**

**Figure S5. Identification of key functional domains in ITGB1 that mediate TNBC tumor development.**

**(A)** FACS plots showing the expression of ITGB1 in Ctrl-KO or *Itgb1*-KO 4T1 cells with or without the introduction of ITGB1-ΔICD, ITGB1-ΔETD, or ITGB1-ΔICD.ETD. ITGB1 truncations were HA-tagged and were detected using an anti-HA antibody. (**B**) FACS gating strategy and quantification showing the percentage of immune cells (CD45^+^), CD11b^+^ myeloid cells (CD45^+^CD11b^+^), and F4/80^+^TAM (CD45^+^CD11b^+^Ly6c^-^Gr-1^low^F4/80^+^) in tumor tissues from 4T1-engrafted Rag2^-/-^, γc^-/-^ mice. (**C-D**) Tumor volume measurement in 4T1-engrafted Rag2-/-, γc-/- mice. *n* = 5 mice; two-way RM ANOVA with multiple comparisons. (**E**) *In vitro* proliferation of Ctrl-KO 4T1 cells with or without the introduction of ITGB1-ΔICD and ITGB1-ΔETD. *n* = 3; two-way RM ANOVA with multiple comparisons. (**F**) Examination of the viability of Ctrl-KO 4T1 cells with or without the introduction of ITGB1-ΔICD and ITGB1-ΔETD, by Annexin V and Sytox blue staining. Representative FACS plots (left) and quantification of viable cells (right) are shown. *n* = 3; one-way RM ANOVA with multiple comparisons. (**G**) Growth of tumor developed by Ctrl-KO + empty vector, Ctrl-KO + *ITGB1*.ΔICD, Ctrl-KO + *ITGB1* MDA-MB-231A cells in Rag2-/-, γc-/- mice. *n* = 5 mice; two-way RM ANOVA with multiple comparisons test. (**H**) FACS gating strategy and quantification showing the percentage of CD11b^+^ myeloid cells and F4/80^+^ TAM of tumor tissues from MDA-MB-231A engrafted Rag2-/-, γc-/- mice. *n* = 5 mice. One-way ANOVA with multiple comparisons test. ns, no significance, **P* < 0.05, ****P* < 0.001, *****P* < 0.0001. Data are represented as mean ± SD.

**
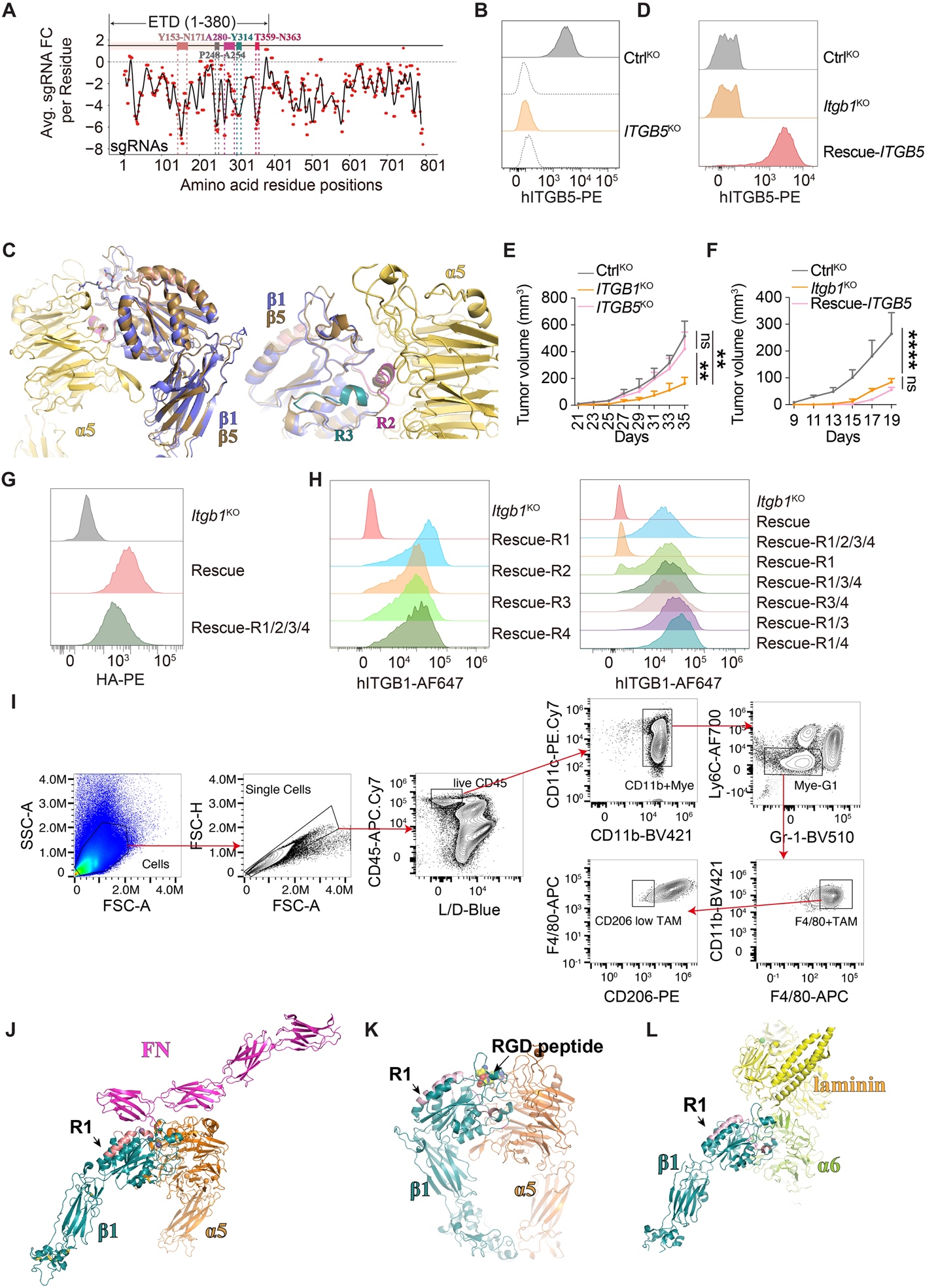
**

**Figure S6.** **Identify the key functional domains in ITGB1.**

(**A**) *In vivo* CRISPR screen to identify ITGB1 functional domains. Individual sgRNAs are shown by red dots and are mapped to amino acid locations. Depletion of sgRNAs was calculated based on the abundance from *in vivo* lung metastatic cells and *in vitro* cultured control cells. The black line shows the smoothed signal levels. (**B**) FACS plots showing the expression of hITGB5 in Ctrl-KO and *ITGB5*-KO MDA-MB-231A cells. (**C**) Structures of β1 (slate) and β5 (brown) are well superimposed. The structure of β5 was predicted by AlphaFold3. (**D**) FACS plots showing the expression of hITGB5 in Ctrl-KO, *Itgb1*-KO, *ITGB5*-Rescue (*Itgb1*-KO with re-introduced hITGB5) 4T1 cells, as detected by anti-hITGB5 antibody. (**E**-**F**) Tumor volume measurement in MDA-MB-231A (E) or 4T1-engrafted (F) Rag2^-/-^, γc^-/-^ mice. *n* = 6 mice; two-way RM ANOVA with multiple comparison test. (**G**) FACS plots showing the expression of ITGB1 in Ctrl-KO, *Itgb1*-KO, *ITGB1*-Rescue-R1/2/3/4 (*Itgb1*-KO with re-introduced HA-tagged hITGB1-R1/2/3/4) 4T1 cells, as detected by anti-HA antibody. (**H**) FACS plots showing the expression of hITGB1 (with one to four regions of R1-R4 swapped) on 4T1 cells, as detected by anti-hITGB1 antibody. (**I**) FACS gating strategy and quantification showing the percentage of immune cells (CD45^+^), CD11b+ myeloid cells (CD45^+^CD11b^+^), and F4/80^+^TAM (CD45^+^CD11b^+^Ly6c^-^Gr-1^low^F4/80^+^) in tumor tissues from 4T1-engrafted Rag2^-/-^, γc^-/-^ mice. (**J-K**) Structure of the human α5β1 ECD in complex with the RGD peptide (PDB ID 3VI4) and FN (PDB ID 7NWL). (**L**) Structure of the human α6β1 ECD in complex with the laminin peptide (PDB ID 7CEC).

**
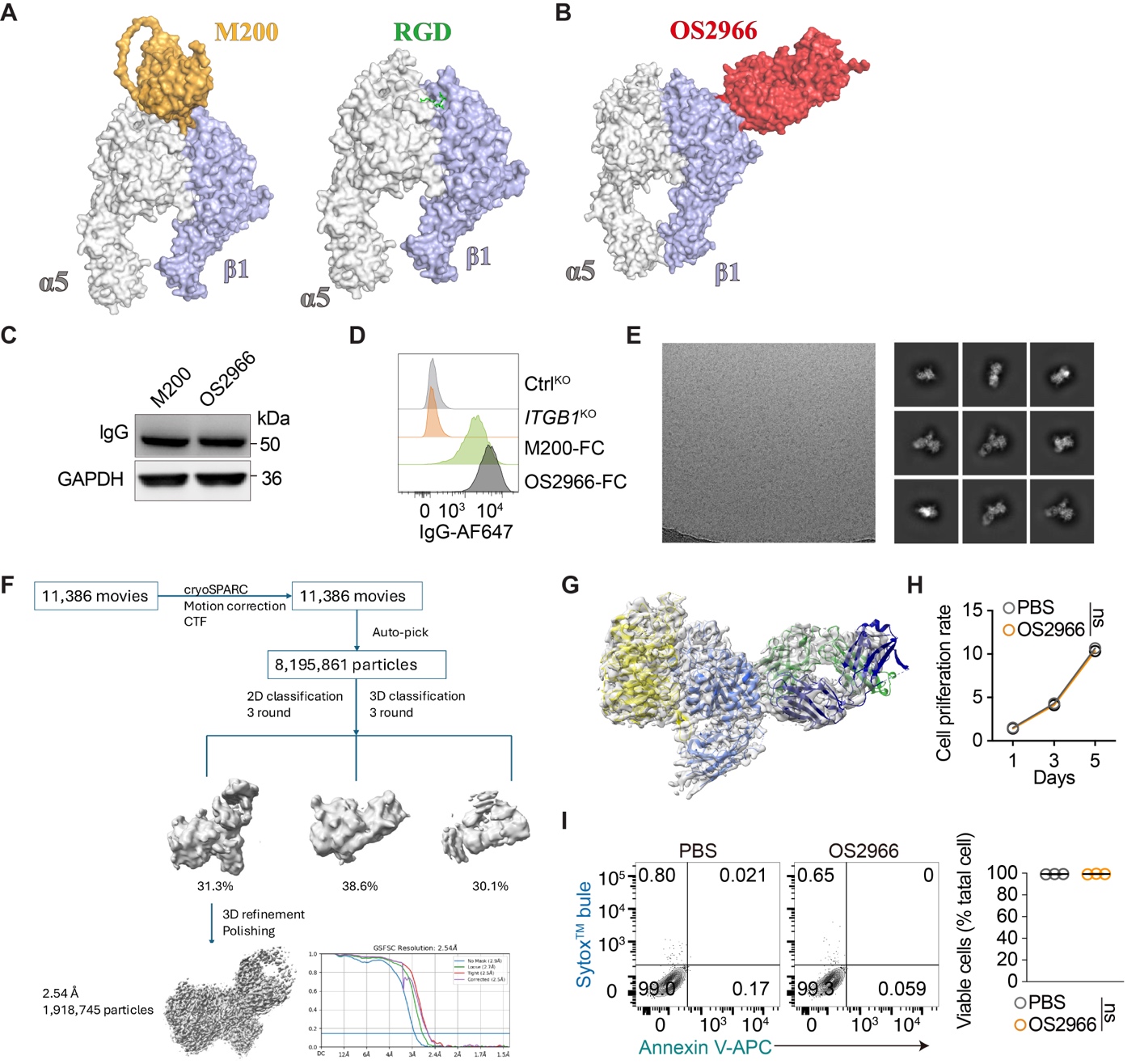
**

**Figure S7. Structure and expression of ITGB1 antibodies.**

(**A**) Structure of the human α5β1 ECD in complex with the M200 antibody (orange) and RGD (PDB ID 3VI4) (green). (**B**) Structural model of the human α5β1 ECD in complex with OS2966 (red). (**C**) Immunoblots of MDA-MB-231A cells expressing the scFv of M200 and OS2966 antibodies. (**D**) FACS plots showing the binding of secreted Fc-conjugated scFv of M200 and OS2966 to MDA-MB-231A cells. (**E**) Representative cryo-EM micrograph and 2D class averages. (**F**) Cryo-EM image processing workflow and Gold-standard Fourier shell correlation (FSC) curve showing an overall resolution is 2.54 Å at FSC = 0.143. (**G**) Cryo-EM of the human α5β1 ECD in complex with OS2966 Fab. (**H**) Examination of the *in vitro* proliferation of MDA-MB-231A cells treated with PBS or OS2966. *n* = 3; two-way RM ANOVA with multiple comparison test. (**I**) Examination of the viability of MDA-MB-231A cells treated with PBS or OS2966 by Annexin V and Sytox blue staining. Representative FACS plots (left) and quantification of viable cells (right) are shown. *n* = 3; unpaired *t*-test. In all figures, ns, no significance; Data are represented as mean ± SD.

**
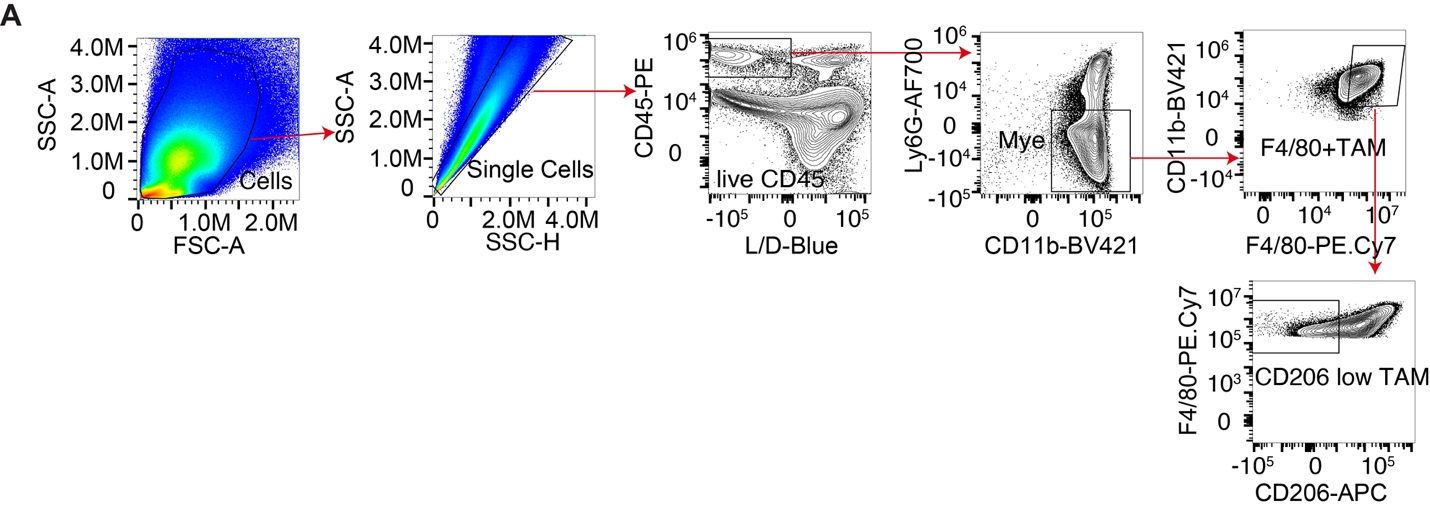
**

**Figure S8. Flow cytometry phenotyping of MDA-MB-231A tumor tissues.**

(**A**) FACS gating strategy and quantification showing the percentage of immune cells (CD45^+^), CD11b^+^ myeloid cells (CD45^+^CD11b^+^), and F4/80^+^TAM (CD45^+^CD11b^+^Ly6G^-^F4/80^+^) in tumor tissues from MDA-MB-231A-engrafted Rag2^-/-^, γc^-/-^ mice. Data are represented as mean ± SD.

**
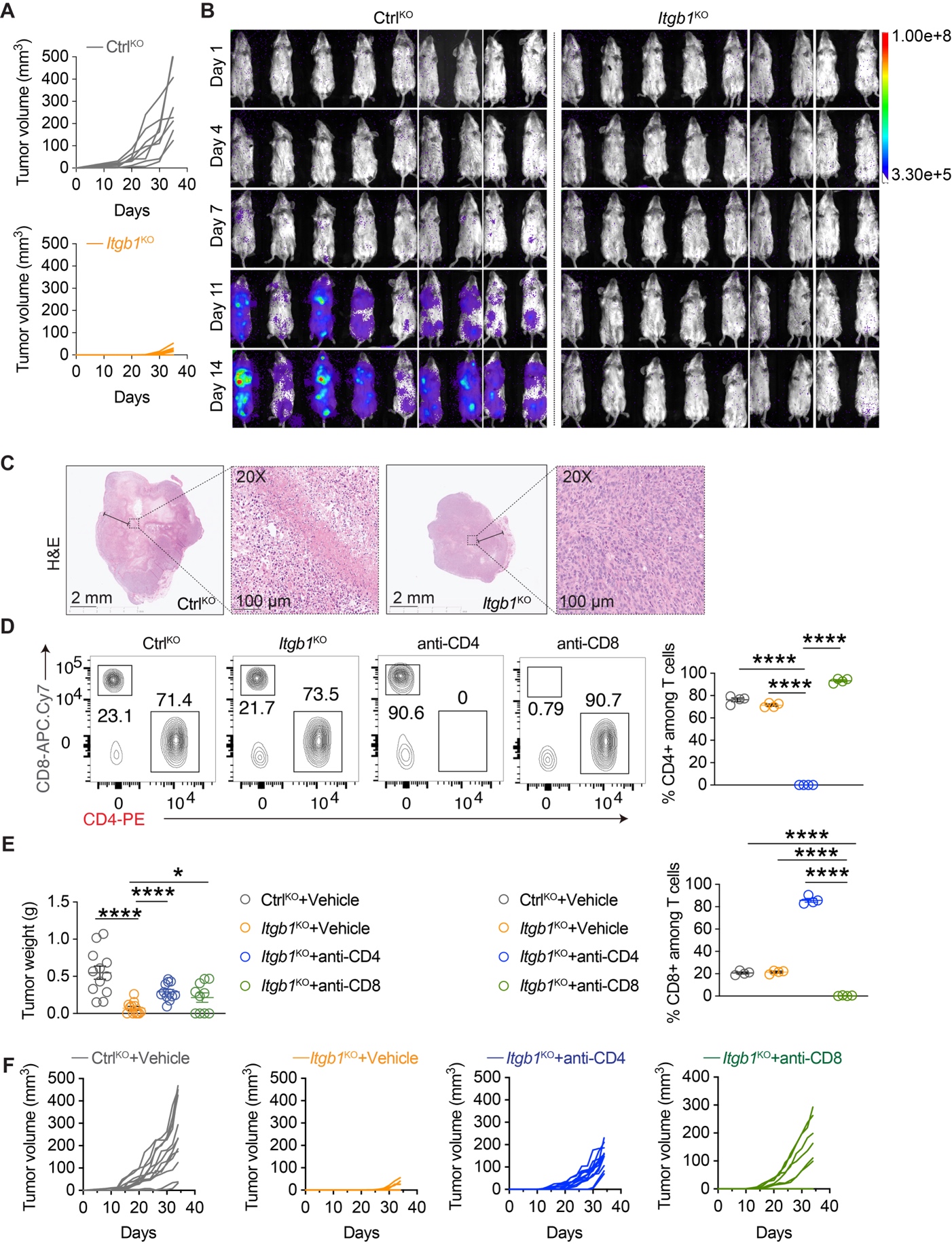
**

**Figure S9.** **Evaluation of ITGB1’s pro-tumor activities in immunocompetent mice and immunohistochemical staining.**

(**A**) Tumor growth curve of individual mice in Figure 6A. *n* = 8 (Ctrl-KO) or 10 (*Itgb1*-KO) mice per group. (**B**) Bioluminescence imaging of BALB/c mice intravenously inoculated with Ctrl-KO or *Itgb1*-KO 4T1 cells. (**C**) Representative photomicrographs of H&E staining in tumor tissues from Ctrl-KO or *Itgb1*-KO 4T1-engrafted BALB/c mice. **(D)** Representative FACS plots and quantification showing CD4 (CD45^+^CD3^+^CD4^+^) or CD8 (CD45^+^CD3^+^CD4^+^) T cells in tumor tissues from Ctrl-KO or *Itgb1*-KO 4T1-engrafted BALB/c mice, which were treated with CD4- or CD8-neutralizing antibodies. *n* = 4 mice; one-way ANOVA with multiple comparisons. (**E-F**) Tumor weight (E) and tumor growth curve of individual mice (F) of Ctrl-KO or *Itgb1*-KO 4T1-engrafted BALB/c mice; Mice received treatments with control vehicles or CD4- or CD8-neutralizing antibodies. *n* = 12 (Ctrl-KO + Vehicle), 12 (*Itgb1*-KO + Vehicle), 12 (*Itgb1*-KO + anti-CD4), or 10 (*Itgb1*-KO + anti-CD8) mice; two-way ANOVA with multiple comparisons. In all figures, ns, no significance, **P* < 0.05, *****P* < 0.0001; Data are represented as mean ± SD.


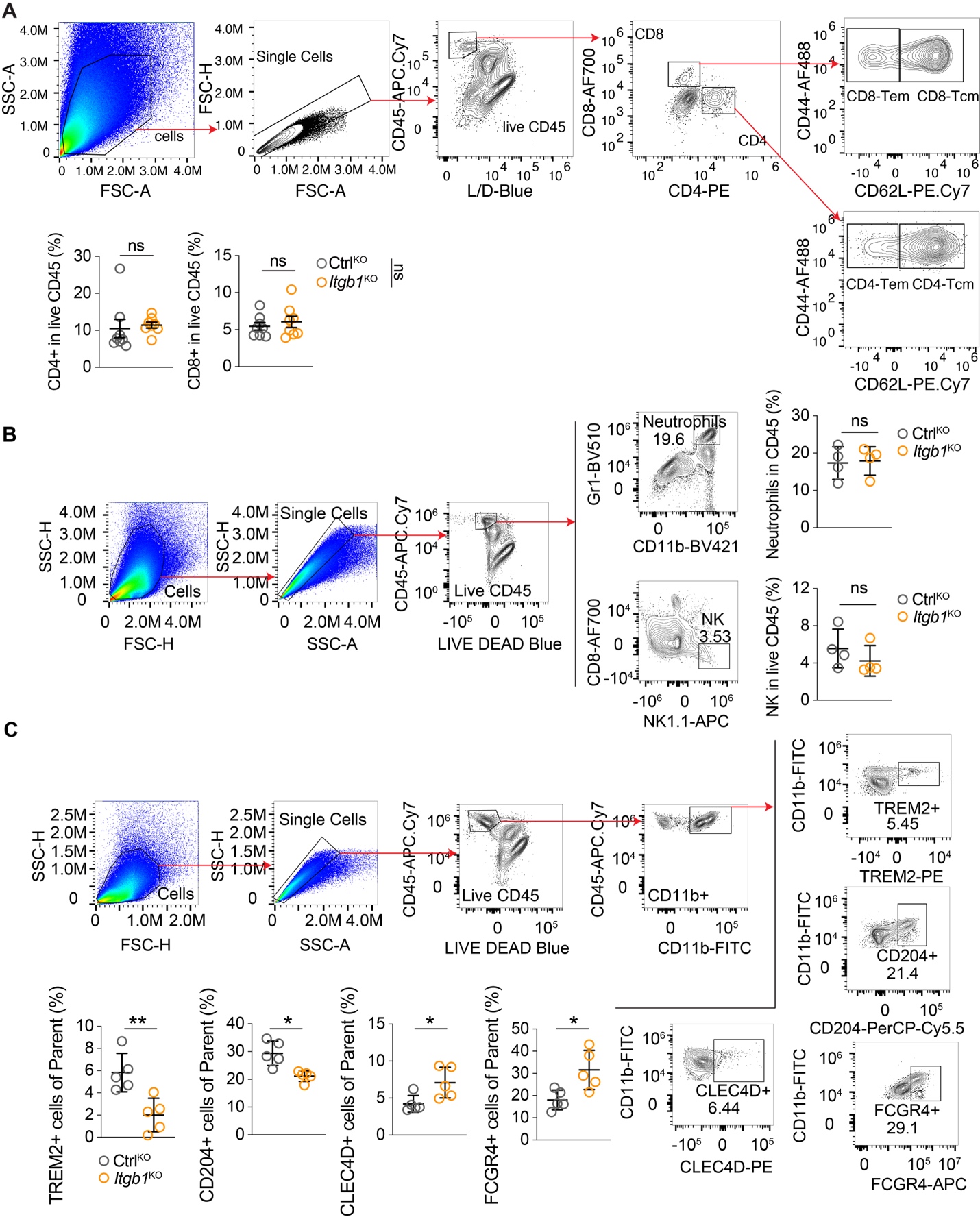


**Figure S10. Evaluation of ITGB1’s role in T cell infiltration and adaptive immune response.**

(**A**) FACS gating strategy and quantification showing the percent of immune cells (CD45^+^), CD4 T cells (CD45^+^CD4^+^), or CD8 T cells (CD45^+^CD8^+^) in tumor tissues from Ctrl-KO or *Itgb1*-KO 4T1-engrafted BLAB/c mice. *n* = 8 mice; unpaired *t*-test. (**B**) FACS gating strategy and quantification showing the percentage of Neutrophils or NK cells within the CD45^+^ cells of tumor tissues from Ctrl-KO or *Itgb1*-KO 4T1 engrafted BALB/c mice. *n* = 4 mice; unpaired t-test. (**C**) FACS gating strategy and quantification showing the percentage of TREM2^+^, CD204^+^, CLEC4D^+^, FCGR4^+^ cells within the CD45^+^CD11b^+^ myeloid cells of tumor tissues from Ctrl-KO or *Itgb1*-KO 4T1 engrafted BALB/c mice. *n* = 5 mice; unpaired t-test. In all figures, ns, no significance, **P* < 0.05, ***P* < 0.01; Data are represented as mean ± SD.


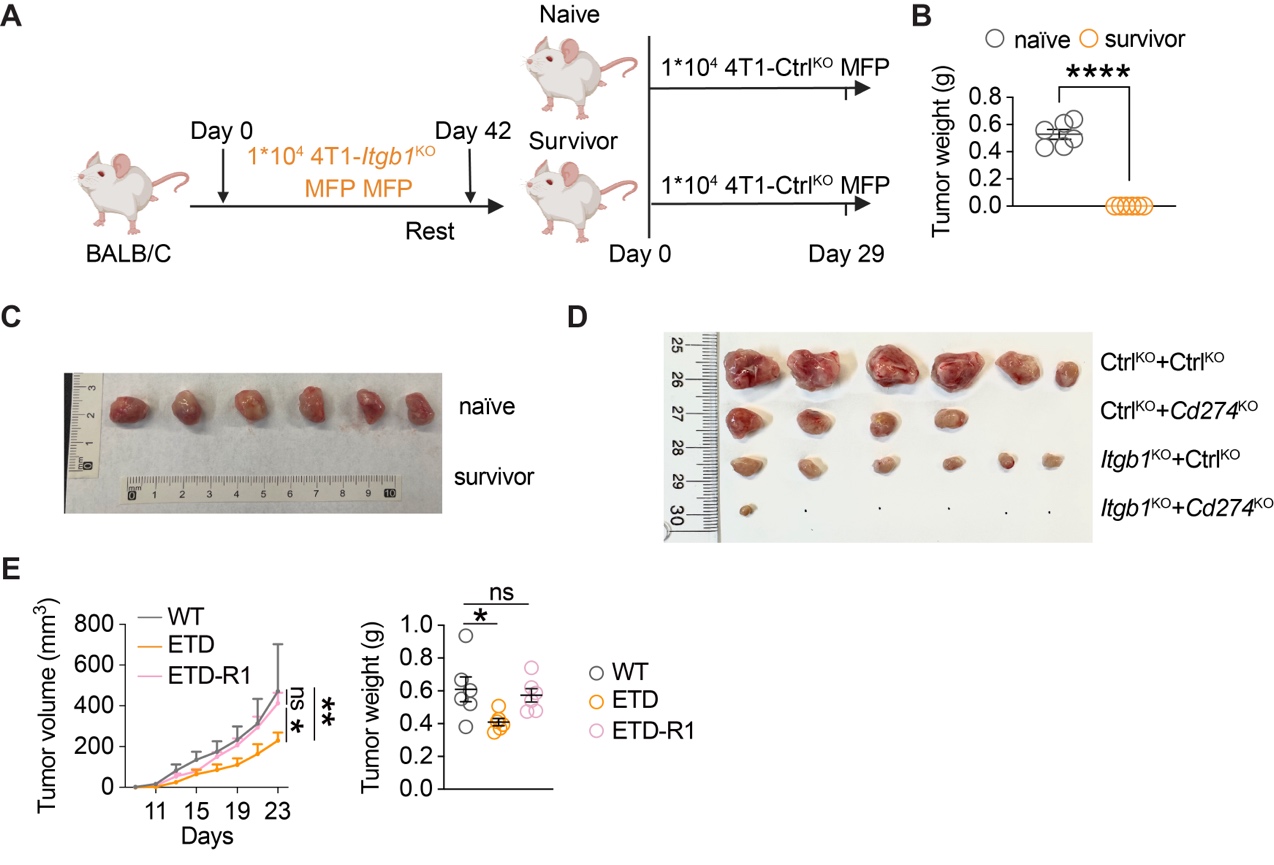


**Figure S11. Evaluation of ITGB1’s role in adaptive immune response.**

(**A**) A schematic showing the rechallenge strategy. **(B-C)** Weight (B) and image (C) of tumors developed by 4T1 cells; Day 29 after reintroducing Ctrl-KO 4T1 cells into naïve mice or mice that failed to engraft *Itgb1*-KO 4T1 tumors. *n* = 6 mice; unpaired *t*-test. (**D**) Image of tumors developed from Ctrl-KO and *Cd274*-KO 4T1-Ctrl-KO or *Itgb1*-KO cells in BALB/c mice. (**E**) Tumor volume measurement (left) and tumor weight (right) in 4T1-engrafted BALB/c mice. *n* = 6 mice; two-way RM ANOVA (left) or one-way ANOVA (right) with multiple comparisons. In all figures, ns, no significance, **P* < 0.05, *****P* < 0.0001; Data are represented as mean ± SD.
